# The role of the mucin-glycan foraging *Ruminococcus gnavus* in the communication between the gut and the brain

**DOI:** 10.1080/19490976.2022.2073784

**Published:** 2022-05-17

**Authors:** Erika Coletto, Dimitrios Latousakis, Matthew G. Pontifex, Emmanuelle H. Crost, Laura Vaux, Estella Perez Santamarina, Andrew Goldson, Arlaine Brion, Mohammad K. Hajihosseini, David Vauzour, George M Savva, Nathalie Juge

**Affiliations:** a Gut Microbes and Health Institute Strategic Programme, Quadram Institute Bioscience, Norwich NR4 7UQ, UK; b Norwich Medical School, Biomedical Research Centre, University of East Anglia, Norwich Research Park, Norwich, NR4 7TJ, UK; c School of Biological Sciences, University of East Anglia, Norwich Research Park, Norwich, NR4 7TJ, UK

**Keywords:** Gut-brain axis, *Ruminococcus gnavus*, gut microbiota, human gut symbiont, neurogenesis, sialic acid, intestinal mucus, gnotobiotic mice, mucin glycosylation, metabolite, cognitive function

## Abstract

*Ruminococcus gnavus* is a prevalent member of the human gut microbiota, which is over-represented in inflammatory bowel disease and neurological disorders. We previously showed that the ability of *R. gnavus* to forage on mucins is strain-dependent and associated with sialic acid metabolism. Here, we showed that mice monocolonized with *R. gnavus* ATCC 29149 (*Rg*-mice) display changes in major sialic acid derivatives in their cecum content, blood, and brain, which is accompanied by a significant decrease in the percentage of sialylated residues in intestinal mucins relative to germ-free (GF) mice. Changes in metabolites associated with brain function such as tryptamine, indolacetate, and trimethylamine *N*-oxide were also detected in the cecal content of *Rg*-mice when compared to GF mice. Next, we investigated the effect of *R. gnavus* monocolonization on hippocampus cell proliferation and behavior. We observed a significant decrease of PSA-NCAM immunoreactive granule cells in the dentate gyrus (DG) of *Rg*-mice as compared to GF mice and recruitment of phagocytic microglia in the vicinity. Behavioral assessments suggested an improvement of the spatial working memory in *Rg*-mice but no change in other cognitive functions. These results were also supported by a significant upregulation of genes involved in proliferation and neuroplasticity. Collectively, these data provide first insights into how *R. gnavus* metabolites may influence brain regulation and function through modulation of granule cell development and synaptic plasticity in the adult hippocampus. This work has implications for further understanding the mechanisms underpinning the role of *R. gnavus* in neurological disorders.

## Introduction

The gut microbiota plays an essential role in human health and disease.^1,2^ An alteration in the structure and function of the human gut microbiota is associated with intestinal and extraintestinal diseases including neurodegenerative diseases, stress, anxiety disorders, and neurodevelopmental dysfunctions.^3,4^ In the colon, the gastrointestinal (GI) tract is covered by a mucus bilayer, with the outer layer harboring gut microbes, while the inner layer protects the underlying epithelium from luminal contents and bacterial invasion.^5,6^ Mucin glycosylation plays a critical role in maintaining a homeostatic relationship between the gut microbiota and the host, with terminal epitopes such as fucose or sialic acid being a preferential nutritional and adhesion target for bacteria.^7,8^ *Ruminococcus gnavus*, belonging to the Lachnospiraceae family in the Firmicutes phylum, is a prevalent human gut symbiont and an early colonizer of the infant gut,^9^ which persists throughout adulthood and is one of 57 bacterial species present in more than 90% of individuals.^10^ We previously showed that the adaptation of *R. gnavus* to the gut is strain-dependent and associated with its capacity to forage on mucin glycan epitopes such as sialic acid, fucose, or blood group antigens in the mucus niche.^11–14^ The most common forms of sialic acid are N-acetylneuraminic acid (Neu5Ac) and its hydroxylated form N-glycolylneuraminic acid (Neu5Gc),^9,10^ while further modifications at the hydroxyl or amine group of the sialic acid give rise to many different sialic acid types.^11,12^ We showed that *R. gnavus* ATCC 29149 possesses a unique sialic acid metabolism pathway among the gut microbiota through the production of the 2,7-anhydro-Neu5Ac derivative.^15^

A growing number of metagenomics studies are reporting an association between *R. gnavus* and a range of diseases including inflammatory bowel disease (IBD),^16,17^ and inflammatory autoimmune disease, such as lupus erythematosus.^18^ Altered levels of *R. gnavus* have also been reported in patients suffering from general anxiety disorders,^19,20^ migraine,^21^ depression,^22^ and attention deficit hyperactive disorder (ADHD),^23^ although a causal effect remains to be demonstrated. It is well established that anxiety-related behaviors, stress, and depression can affect neurogenesis by altering the proliferation and differentiation of immature granule cells and ultimately the generation of new neurons,^24,25^ and studies in gnotobiotic mouse models have demonstrated the role of the gut microbiota in this process.^26,27^ The lack of gut microbiota in germ-free (GF) mice results in an impairment of neurogenesis, leading to deficit in the learning process,^28^ while the reconstitution of the gut microbiota in antibiotic-treated mice was shown to promote the recovery of neurons production in the hippocampus.^29^ Neurogenesis plays a pivotal role in the postnatal brain for the neuronal plasticity and formation of new synapses.^30,31^ PSA-NCAM (polysialic acid attached to the neural cell adhesion molecule) is required during embryonic and adult neural progenitor cell development for the correct signaling transmission in the synaptogenesis process.^32–34^ In line with its function, a dysregulation of PSA-NCAM has been observed in several brain disorders such as schizophrenia, bipolar disorder,^35,36^ and Parkinson’s disease.^37^ The formation of new neurons in the subgranular zone (SGZ) of the hippocampus is a tightly regulated process that sees the development and expansion of progenitor cells (GFAP^+^, Nestin^+^, and Sox2^+^) to differentiation in immature cells (PSA-NCAM^+^, Doublecortin^+^, NeuroD1^+^, and Prox1^+^) and maturation into granule neurons (Calbindin^+^, Prox1^+^, Neun^+^, and NeuroD1^+^), the only type of neurons in the hippocampus.^38,39^ Microglia cells also support adult neurogenesis, by modulating the structure, the synapses, and the pruning of the new-born neurons.^40^ In a healthy brain, phagocytic microglia rapidly and efficiently remove dying or damaged neural proliferating cells in a non inflammatory fashion, as part of normal neurodevelopment.^41,42^

How gut microbes influence brain function and neurogenesis is a matter of extensive investigations, and accumulating evidence suggests that microbial metabolites, in particular, short chain fatty acids (SCFAs), play a significant role in modulating neuronal differentiation and microglia cell activation in health and diseased states.^43,44^ GF and gnotobiotic mice have been invaluable models for advancing our mechanistic understanding of how gut microbes influence host response and the role of gut microbes in intestinal homeostasis and for establishing causal links between specific bacterial species and disease phenotypes.^45^ Studies in GF mice showed that the brain is affected in the absence of microbiota, including blood brain barrier (BBB) permeability, microglia, neurogenesis, and synaptic plasticity.^46^ Here, we used gnotobiotic mice to investigate the role of *R. gnavus* ATCC 29149 in the communication between the gut and the brain. We determined the effect of *R. gnavus* monocolonization on intestinal mucin glycosylation, sialic acid derivatives and metabolites and the impact on the PSA-NCAM^+^ granule cells and microglia population inhabiting the neurogenic area of the hippocampus. Furthermore, we determined the effect of *R. gnavus* on synaptic plasticity as well as on the spatial working and recognition memory, learning and reference memory and anxiety-related behaviors of the mice.

## Results

### *R.* *gnavus* colonization affects mucin sialylation, sialic acid derivatives and other brain function-associated metabolites

In this study, GF mice were monocolonized with *R. gnavus* ATCC 29149 and fed a Neu5Ac-free diet (**Table S1**) for a 14-day colonization period, following diet acclimation. The mice were effectively colonized with 1 × 10^8^ cells/mg of feces at day 7, and colonization remained at the same level until day 14 as shown by qPCR analysis using both universal and *R. gnavus* 16S-specific primers (**Fig. S1**).

**Figure 1.**
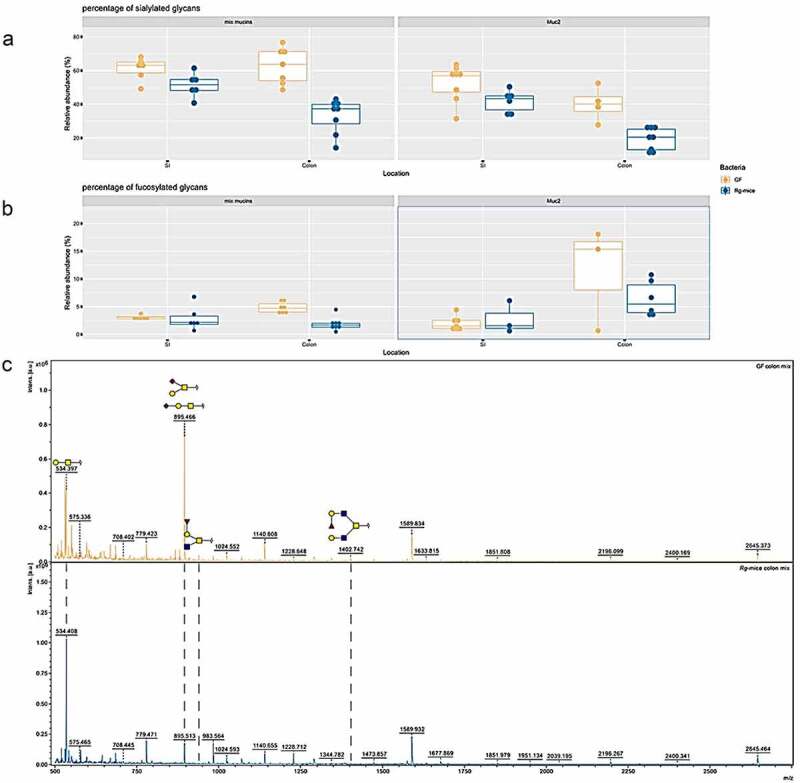
Intestinal mucin glycosylation analysis of GF and *Rg*-mice. Percentage of (a) sialylated or (b) fucosylated glycans in the different mucin fraction from GF (yellow) and *Rg*-mice (blue). Each dot represents a technical replicate. (c) Characteristic mass spectra of glycans from mix mucins from colon of GF (yellow; top) or *Rg*-mice (blue; bottom).

We first investigated the glycosylation profile of intestinal mucins upon colonization of the mice with *R. gnavus* ATCC 29149. Glycans from Muc2 and mix mucins purified from mucus of the small intestine (SI) and colon of GF and *Rg*-mice were analyzed by MALDI-ToF following their release by reductive β-elimination and permethylation. The mass spectra indicate a low degree of glycosylation of Muc2 in GF mice (data not shown), in agreement with previous studies.^47^ To measure the percentage of sialylated and fucosylated glycans across different areas and groups, samples from all mice within each group were pooled and repeated measurements were taken (Figure 1A-1B). Here, the distribution of these technical replicates across groups is shown. Differences were observed between SI and colonic Muc2, with sialylation being higher (~53% ± 11%) in the SI as compared to the colon (~40% ± 10%) in both mouse groups (Figure 1). In GF mice, no differences were observed in the sialylation of mix mucins between the two intestinal regions (61% ± 7% and 63% ± 11% in SI and colon, respectively) (Figure 1A). Furthermore, the fucosylated structures were shown to be higher in the colonic Muc2 of GF mice (11% ± 9%) as compared to the SI (2% ± 1%) (Figure 1B), while no major differences were observed in the fucosylation of mix mucins (3% ± 0.4% and 5% ± 1% in the SI and colon, respectively) (Figure 1B). *Rg*-mice displayed a lower percentage of sialylation of mix mucins in the colon as compared to the GF mice (~33% ± 10% vs ~63% ± 11%, respectively). This difference was largely caused by the reduction in the relative abundance of sialyl-T antigen and sialyl-Core 1 glycans and the concurrent increase of T-antigen in *Rg*-mice compared to GF mice, as shown by the reduction of the 895 m/z peak on the MS chromatograms and increase of the peak at m/z 534 (Figure 1C). The percentage of sialylation in mix mucins in the SI only showed a small reduction in *Rg*-mice compared to GF mice (~51% ± 7% vs ~61% ± 7%, respectively) (Figure 1 A). The sialylation of Muc2 in the SI was slightly decreased in *Rg*-mice as compared to GF mice from 53% ± 11% to 42% ± 7%, as did in the colon, from 40% ± 10% to 19% ± 7% (Figure 1A). A lower percentage of fucosylation of mix mucins in the colon was observed in *Rg*-mice as compared to GF mice (~5% ± 1% vs ~2% ± 1%, respectively) (Figure 1B), as illustrated by the absence of the fucosylated glycan peaks at 954 m/z and 1402 m/z in *Rg*-mice relative to GF mice (Figure 1C). No differences were detected in the percentage of fucosylation of mix mucins or Muc2 in the SI between the two mouse groups (Figure 1B).

**Figure 2.**
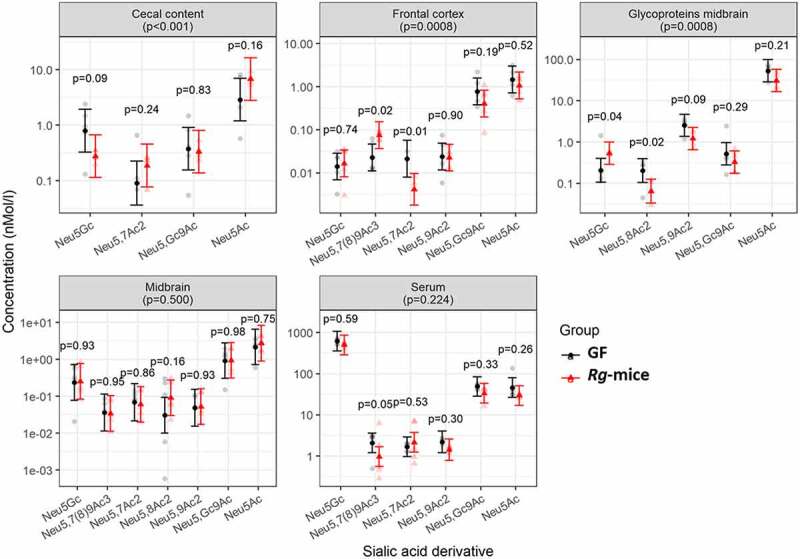
Concentrations of sialic acid derivatives from different areas of GF and *Rg*-mice. Sialic acid derivative levels in GF mice and *Rg*-mice as determined by HPLC, expressed in nmol/L in cecal content, frontal cortex, midbrain, and serum. P-values in panel captions correspond to the test of whether colonization significantly affects the sialic acid profile anywhere within that region, calculated as a simultaneous test of the main effect of the group and the interaction between group and specific sialic acid. Specific p-values correspond to individual comparisons shown, derived from the statistical model described in the text.

Given the importance of sialic acid metabolism in the ability of *R. gnavus* ATCC 29149 to colonize the gut, we next investigated the nature and levels of sialic derivatives in the cecum, brain (free and glycoprotein-bound), and serum of *Rg*-mice and GF mice by HPLC. Estimated concentrations of each derivative in each area (brain, cecum, and serum) and ratios between groups of specific concentrations are shown in Table S2. Regarding the two main sialic acid derivatives, in both mouse types, Neu5Ac concentrations were highest in the brain glycoprotein portion (between ~30 and ~53 nmol/L) and in the serum (between ~29 and ~45 nmol/L), whereas the Neu5Gc concentration was highest in the serum (between ~517 and ~602 nmol/L) as compared to the other regions tested. Neu5,7Ac2, Neu5,8Ac2, Neu5,9Ac2, and Neu5,Gc9Ac derivatives were present only at low levels in the serum (from ~0.09 to ~2 to nmol/L), cecal content (from ~0.09 to ~0.3 nmol/L), and in the glycoprotein from brains (from ~0.2 to ~2.5 nmol/L) of GF and *Rg*-mice. We then compared the pattern of sialic acid derivatives varying between mouse groups. There was no evidence for overall differences in sialic acid derivatives in the serum (p = 0.2242) or midbrain (p = 0.5004) of *Rg*-mice as compared to GF mice, but the overall pattern of sialic acid derivatives in the cecal content (p < .0001), frontal cortex (p = 0.00075) and in the brain glycoproteins (p = 0.0008) did vary significantly between the two groups (Figure 2). In the cecum, the concentration of Neu5Ac in *Rg*-mice was as substantially higher than that of Neu5Gc although not statistically significant (p = 0.160), as compared to the ratio between the two derivatives in GF mice (Figure 2A). In the frontal cortex, the concentration of Neu5,7Ac2 was lower in the *Rg*-mice group (p = 0.015), while Neu5,7(8)9Ac3 was higher (p = 0.023) (Figure 2B). In the midbrain glycoproteins, Neu5Gc concentrations appeared to be higher in the *Rg*-mice (p = 0.042), while in other derivatives, in particular, Neu5,8Ac2 (p = 0.019) appeared lower in *Rg*-mice as compared to GF mice (Figure 2C).

**Figure 3.**
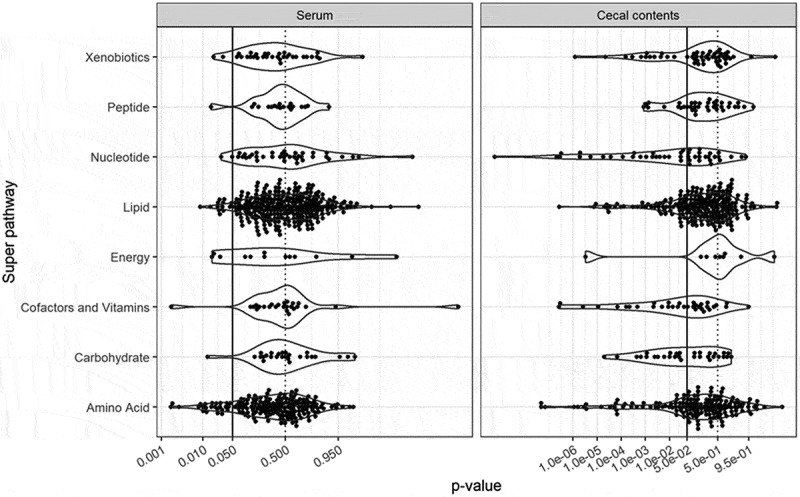
Statistical analysis of the effect of *R. gnavus* monocolonization on metabolites. The distribution of p-values for t-tests of the effect of *R. gnavus* monocolonization on the log-concentration of metabolites was stratified by superpathway and by area (serum and cecal content). Vertical lines correspond to p = 0.5 (dotted line) and p = 0.05 (solid line).

**Figure 4.**
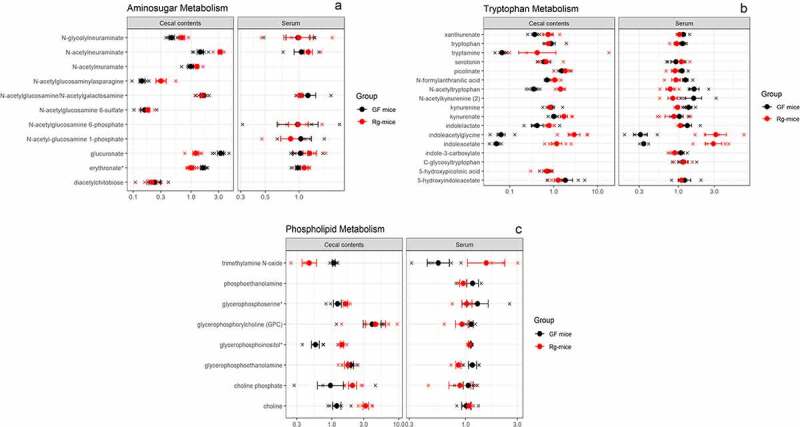
Scaled metabolite concentrations of selected subpathways in cecal contents and serum of GF and *Rg*-mice. Individual data points corresponding to each mouse are shown, along with group means and error bars corresponding to ± 1 standard error for each metabolite.

Untargeted metabolomics were then carried out in order to investigate the range of metabolites affected by the colonization of GF mice with *R. gnavus* ATCC 29149. These analyses led to the identification of 584 compounds of known identity in the cecal content and 611 in the serum of GF and *Rg*-mice. The distribution of p-values for the effect of *R. gnavus* monocolonization on each metabolite, stratified into superpathways, is shown in Figure 3. In the serum, 48 out of 611 (8%) metabolites showed a significant difference at p < 0.05, with only the amino acid superpathway being over-represented with 25 out of 177 metabolites (14%) affected (p-value for the hypergeometric test < 0.001). In the cecal contents, 189 out of 584 (32%) metabolites were significantly different between groups, with superpathways nucleotide (24/46), carbohydrates (16/31), and vitamins/cofactors (17/34) including the most affected compounds (**Table S3)**. Concentrations of specific metabolites corresponding to aminosugar metabolism, phospholid metabolism, and tryptophan metabolism are shown in Figure 4. A significant increase in *N*-acetylneuraminates was detected in the cecal content of *Rg-*mice (p = 0.001; q = 0.008), whereas no difference was observed in the serum of *Rg-*mice as compared to GF mice (p = 0.459) (Figure 4A) in agreement with the targeted analysis of sialic acid derivatives (Figure 2). In addition, several *R. gnavus-*derived metabolites known as modulators of brain function were detected. Tryptamine was detected in the cecal content of *Rg-*mice, while it was not detected in GF mice or in the serum of both groups (Figure 4B). The concentration of the catabolite *N*-acetyltryptophan was significantly higher in the cecal content of *R. gnavus* mice (p < 0.001; q < 0.001), whereas an opposite effect was observed in the serum albeit not statistically significant after correction for multiple testing (p = 0.01; q = 0.479) (Figure 4B). *Rg*-mice showed a significant increase in indolacetate and indolacetylglycine in the cecal content (p < 0.001, q < 0.001 for both compounds) and in the serum as compared to GF mice (Figure 4B). Furthermore, changes in energy metabolism (TCA cycle) in the cecal content of *Rg*-mice relative to GF mice (Figure 3) were accompanied by alterations in metabolites associated with the phospholipid metabolism, such as choline (p = 0.0007; q = 0.007) that is involved in the synthesis of trimethylamine *N*-oxide (TMAO) with increased TMAO levels (p = 0.079; q = 0.6557) in the serum of *Rg*-mice (Figure 4C).

### Colonization with *R.* *gnavus* affects PSA-NCAM^+^ granule cell lineage with no impact on microglia morphology

**Figure 5.**
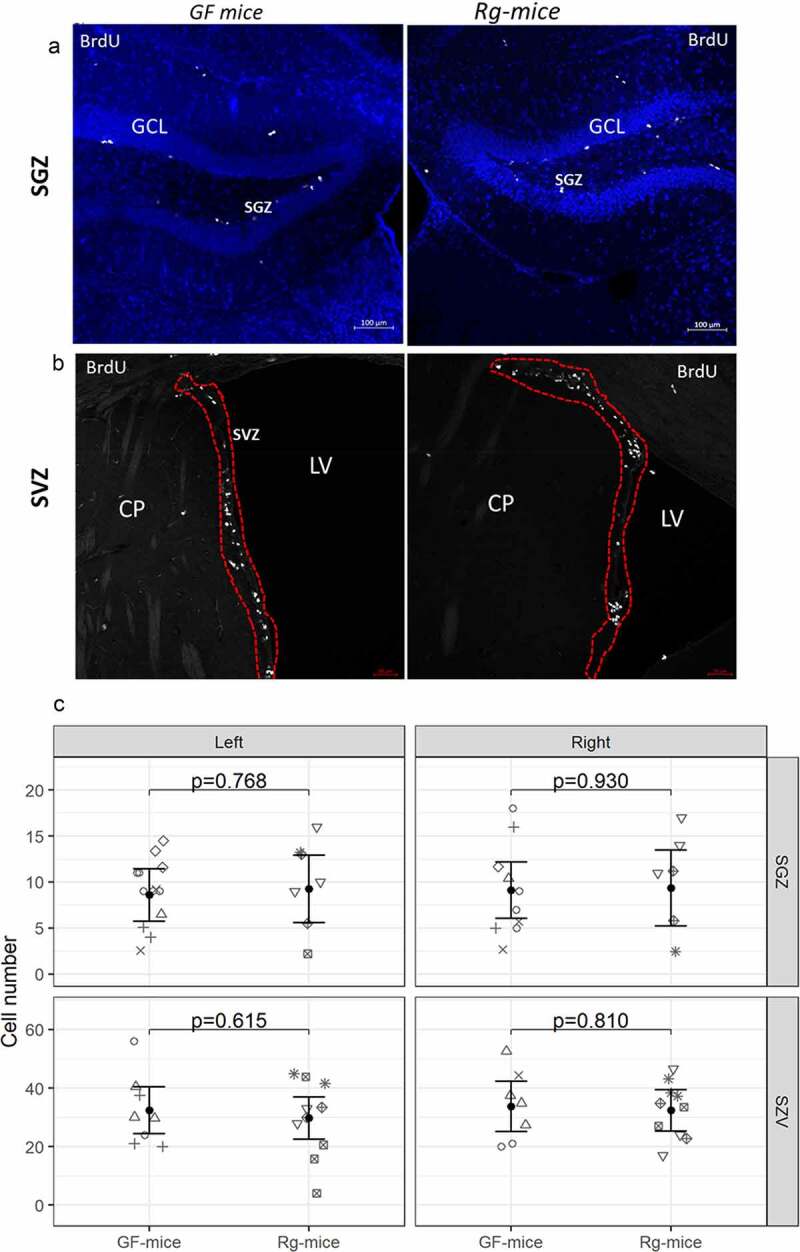
Imaging and quantitative analysis of BrdU incorporation in the SGZ and SVZ of *Rg-*mice and GF mice. (A) BrdU labeling in the DG of GF mice and *Rg*-mice from agar-embedded brain sections. In the DG, the SGZ underlying the granular cell layer (GCL) represents the main region of proliferative activity in both GF and *Rg*-mice. (B) BrdU labeling in the SVZ of GF mice and *Rg*-mice from agar-embedded brain sections. The SZV forms a continuous stream facing the lateral ventricle (LV) (dashed red line) alongside the caudate/putamen nucleus (CP) (lower panel). (C) Analysis of BrDU-positive cells between *Rg-*mice and GF mice. Each symbol corresponds to individual mice.

To determine whether colonization with *R. gnavus* ATCC 29149 could affect adult neurogenesis, we first examined the degree of BrdU incorporation by proliferating neural progenitor cells in the two canonical neurogenic niches of adult rodent brain,^8^ namely, the subgranular zone (SGZ) of the hippocampal dentate gyrus (DG) (Figure 5A) and the lateral subventricular zone (SVZ) of the lateral ventricles (Figure 5B). Briefly after a short BrdU pulse, BrdU-positive cells were quantified in z-stack images (12 slices with 8 µm apart) derived from coronal sections of *Rg*-mice and GF mice. For each animal, 4 to 6 sections were analyzed, and to accurately quantify whole BrdU labeled nuclei in each stack, the first and the last slices surface stacks were excluded from the analysis. There was no difference between *Rg*-mice and GF mice with respect to the total number of BrdU-positive cells in SZV or SGZ (Figure 5C). The number of BrdU-positive cells was significantly lower in the SGZ as compared to SVZ in both groups (p < 0.001) although, in line with previous reports that of the two niches, SVZ contains more proliferating cells.^48^

We next investigated the effect of *R. gnavus* monocolonization on the distribution and morphology of Iba-1-expressing microglia and PSA-NCAM-positive intermediate progenitor cells. For this analysis, coronal brain sections were double immunolabeled with antibodies against these markers. In each mouse brain, between 4 and 7 sections spanning rostrocaudal Bregma co-ordinates −1.58 to −2.54 mm were analyzed, and 5 mice were used per experimental group.

**Figure 6.**
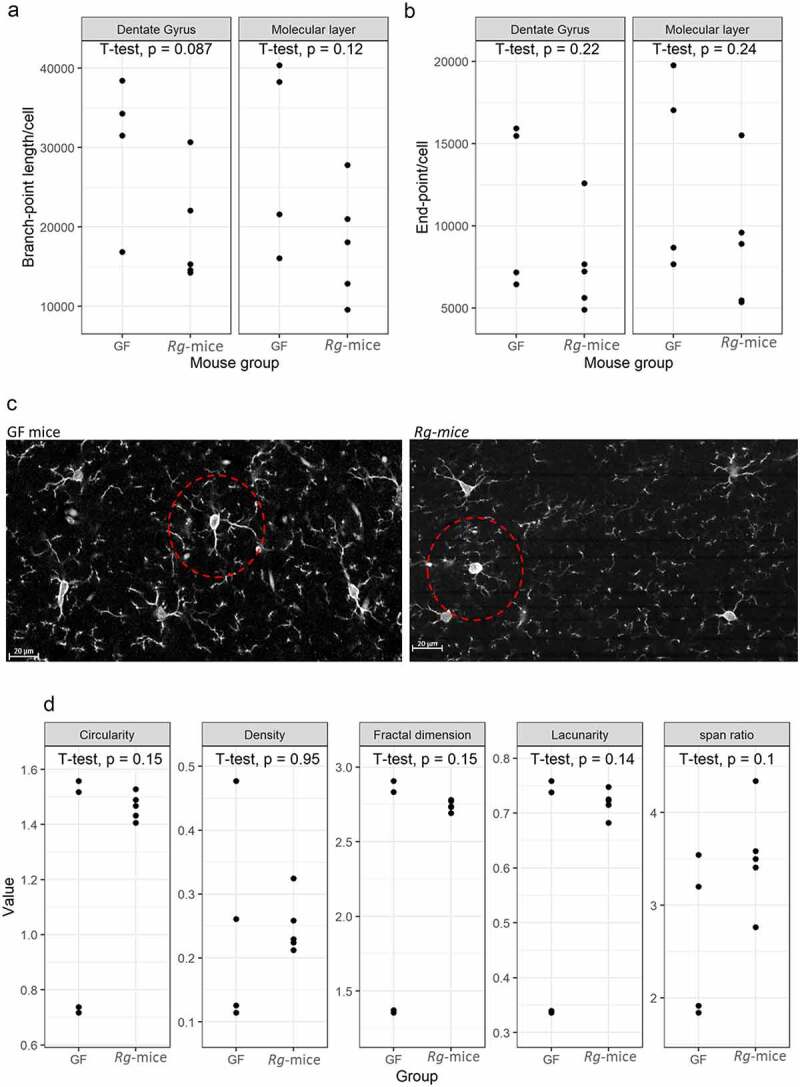
Morphological analysis of microglia in the DG of *Rg*-mice and GF mice. (a) Comparative analysis of the microglia branch-point length in the ML and Hi of GF and *Rg*-mice. (b) Comparative analysis of the microglia endpoints in the ML and Hi of GF and *Rg*-mice. (c) Images showing microglia morphology with enlarged cell soma and shortenings of ramification (dashed red line) in *Rg*-mice as compared to GF mice. (d) Analysis of microglia fractal analysis in GF and *Rg*-mice.

First, we employed skeleton and fractal quantitative analyses to compare potential differences in microglial morphology between *Rg*-mice and GF mice, focusing on the molecular layer (ML) and the hilus (Hi) in the DG regions of hippocampus (**Fig. S2**) The analysis of endpoints and process length data, used to estimate the extent of microglia ramifications, showed no statistically significant differences in process lengths of microglia (branch length/cell; p = 0.090) (Figure 6A) and endpoints lengths (p = 0.22) (Figure 6B) in *Rg-*mice when compared to GF mice. GF mice showed an elongated or rounded cell body with short branches (Figure 6C), and these morphological features were also observed in microglia cells in *Rg*-mice (Figure 6D).

**Figure 7.**
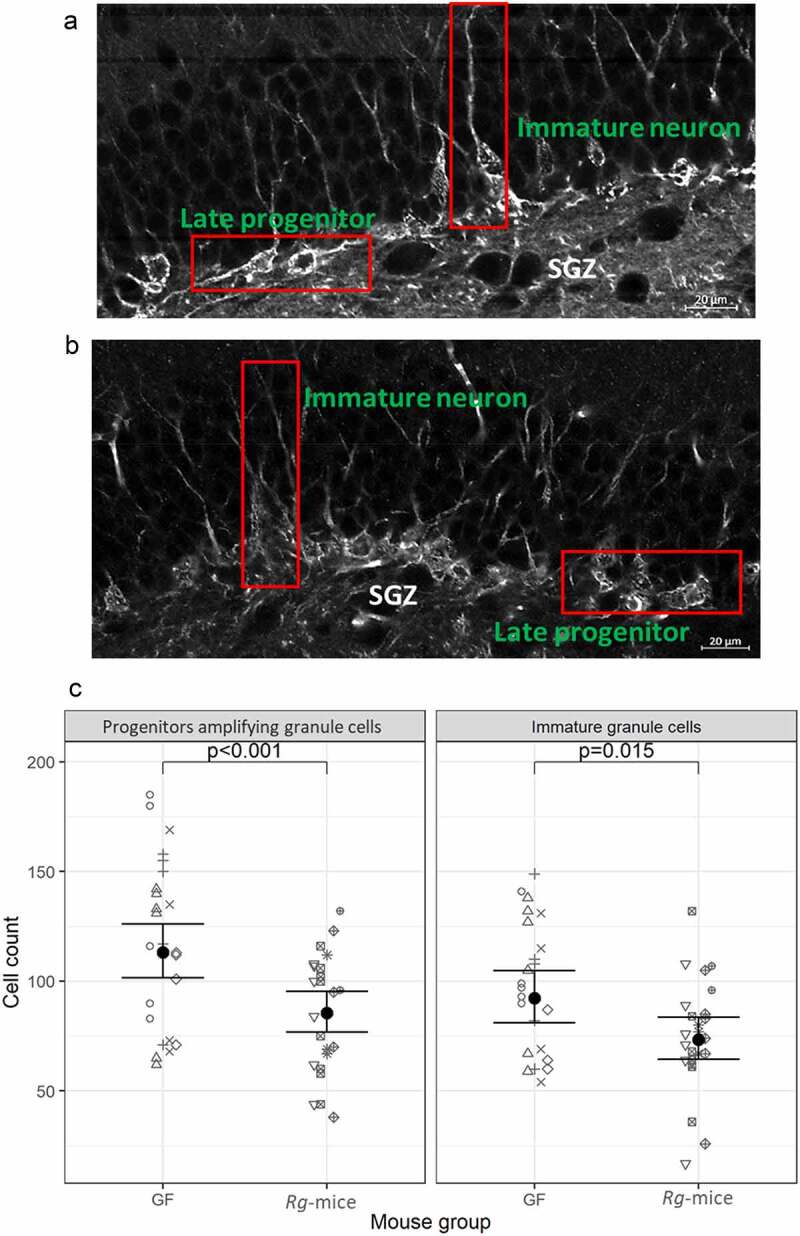
Analysis of PSA-NCAM-positive cells in the SGZ of *Rg*-mice and GF mice. (a) Phenotype and ratio of late progenitor cells/immature granule neurons in the SGZ of *Rg*-mice. (b) Phenotype and ratio of late progenitor cells/immature granule neurons in the SGZ of GF mice is shown in the image. (c) Counts of immature granule cells and progenitor cells in the SGZ of the DG stratified by the mouse group. The gray points represent individual counts with symbols corresponding to individual animals. Marginal means are represented by black dots with 95% confidence intervals shown. Image: screenshot of DG, scale bar: 20 µm. Each symbol corresponds to individual mice.

Next, PSA-NCAM immunoreactivity was determined in Bregma-matched brain sections of *Rg*-mice and GF mice. Using the ImageJ plugin NeuronJ, we quantified PSA-NCAM-positive cells and measured the length of the neurites extending from the immature granule cell soma interspersed in the SGZ of the DG. Although a difference in arborization was not detected between *Rg-*mice and GF mice, a significant alteration in the cell phenotype characterizing the neurogenic pool in the SGZ was observed (Figure 7A/B). *R*g-mice had fewer progenitor cells (ratio = 0.76, 95% CI = 0.66 to 0.96; p = 0.0146) and fewer immature granular cells (ratio = 0.76, 95% CI = 0.65 to 0.88; p = 0.0004) compared to GF mice (Figure 7C).

**Figure 8.**
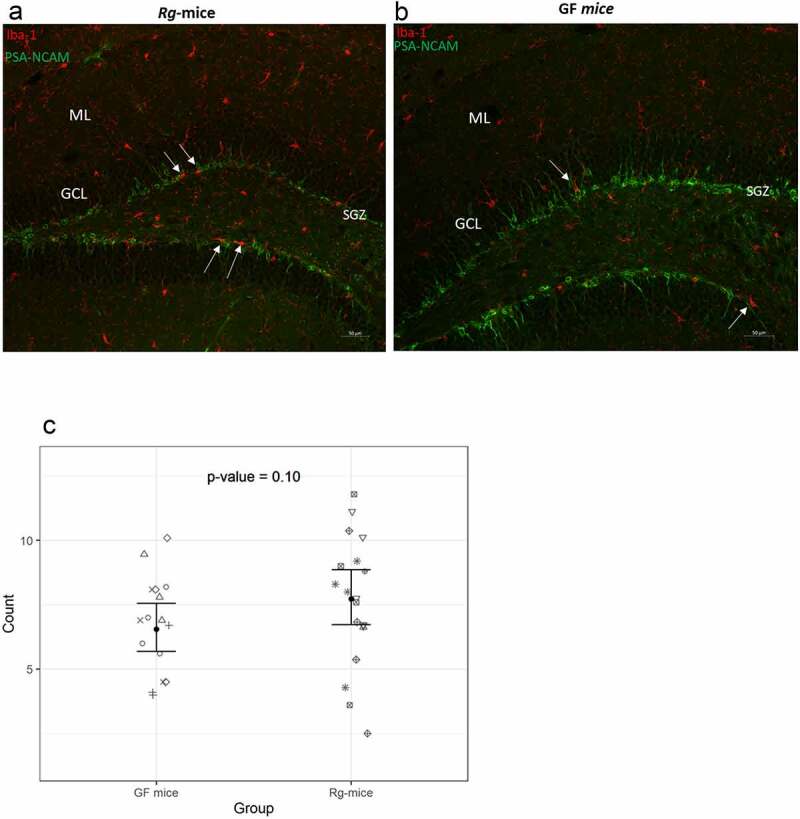
Immunofluorescence microscopy analysis of immature granule cells and microglia in the *Rg*-mice and GF mice hippocampus. (a and b) Immunohistochemistry images showing PSA-NCAM (green) and Iba-1 (red) expression in the DG of *Rg-*mice and GF mice. The double immunolabeling with Iba-1 (red) showed a higher presence of microglial cells (white arrows) in close proximity to the PSA-NCAM-positive cells in the ventral SGZ of (A) *Rg*-mice as compared to (B) GF mice. (C) Number of phagocytic microglial cells (Iba-1-positive) in the SGZ of *Rg*-mice and GF mice. Images: objective 20×, scale bar: 50 µm. Each symbol corresponds to individual mice.

In addition, Iba-1-positive cells were found to be more closely associated with the granule cells in the SGZ of the *Rg*-mice (Figure 8A) as compared to GF mice (Figure 8B), although not statistically significant (ratio = 1.18, 95% CI = 0.97–1.43; p = 0.102) (Figure 8C). Together, these findings suggest that the microglial cells in the SGZ of *Rg*-mice might have an impact on the formation of newborn PSA-NCAM-positive cells in the subgranular area.

### Colonization with *R.* *gnavus* influences the expression of genes involved in synaptic plasticity and proliferation

Having established that mice monocolonized with *R. gnavus* showed a significant decrease of PSA-NCAM^+^ granule cells in the DG, we next investigated how this may relate to the developmental process and synaptic plasticity in the hippocampus of these mice as compared to GF mice. Gene expression analysis was carried out by RT-PCR using validated primers against a panel of 20 target genes and 4 different housekeeping genes (*Gapdh, Tbp, B2m*, and *Hrpt*). *Gapdh* showed the least variability under the study conditions and therefore was used as a reference gene throughout the analyses. The genes of interest were chosen as an indication of synaptic plasticity, neurodevelopment and proliferation (*Bdnf, Creb1, Mapk1, Grinb2, Camk2a, Ncam1, Ki67, Akt1*, and *Arc*); polysialyltranferase activity (*ST8sia2* and *ST8sia4*); formation of tight junction (*ZO-1, Cldn1*, and *Ocln*); granule cells maturation (*Gfap, Prox1*, and *NeuroD1*); and microglia activation and inflammation (*Trem2* and *Nos2*). PSA-NCAM is dynamically regulated by two polysialyltransferase (polySiaT), *ST8sia2* and *ST8sia4*, which catalyze the transfer of a sialic acid residue from its activated sugar nucleotide precursor CMP-Sia to its major acceptor NCAM.^49,50^

**Figure 9.**
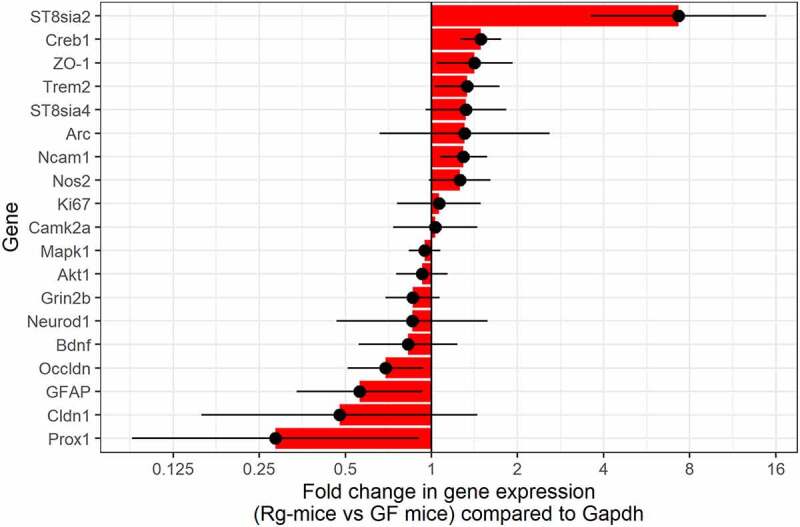
RT-PCR gene expression analysis in the hippocampus of *Rg*-mice and GF mice. Comparative analysis of expressed genes with distinct biological functions. The levels of genes transcripts are shown as fold change compared with the levels of housekeeping gene (Gaphd). All error bars represent mean ± SEM, n = 5 per group.

Fold change of expression for each gene upon colonization with *R. gnavus* is reported with 95% confidence intervals (Figure 9). After adjustment for multiple testing, polysialytransferase (*ST8sia2*, FC = 7.30; FDR-adjusted p-value = 00029) and CAMP-Responsive Element Binding Protein 1 (*Creb1*, FC = 1.49; FDR-adjusted p-value = 0.005) were found to be significantly differentially expressed upon colonization with *R. gnavus*. Together with the observed phenotype of PSA-NCAM^+^ granule cells, these results support the role of *R. gnavus* monocolonization in hippocampal synaptic plasticity and cell development.

*Rg*-mice also showed an upregulation of the neural cell adhesion molecule *(Ncam1 p < 0.02)* phagocytic receptor (*Trem2 p < 0.049*) and zonula occludens 1 (*ZO-1* p < 0.045), whereas genes associated with the proliferative status of granule cells, such as the glial fibrillary acidic protein (*Gfap p < 0.042*) and tight junction occludin (*Ocln* p < 0.033), were found to be significantly downregulated compared to GF mice. The level of expression of the other 12 genes was found to be upregulated (*ST8sia4, Arc*, and *Nos2*) and downregulated (*Cldn1, Prox1, BDNF, NeuroD1*, and *Grin2b*) in *Rg-*mice as compared to GF mice. No other genes were significantly associated with colonization. The levels of expression of *Ki67, Camk2a, Mapk1*, and *Akt1* were comparable between *Rg*-mice and GF mice (Figure 9).

### Effect of *R.* *gnavus* colonization on spatial working memory, recognition memory, and anxiety-related behavior

**Figure 10.**
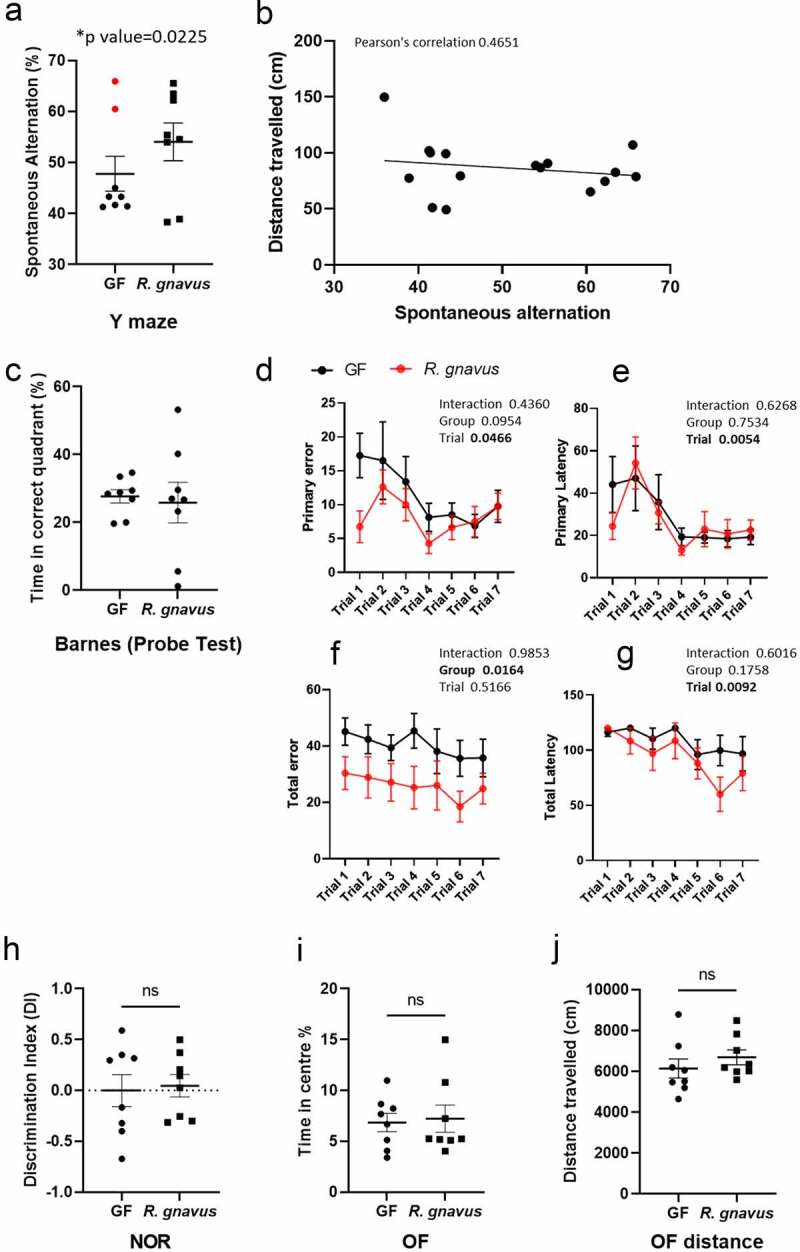
Behavioral tests of cognition in GF and *Rg*-mice. (A) Y-maze spontaneous alternation task in *Rg-mice* when compared to GF mice suggests improved spatial working memory (B), which was not a product of altered mobility. The Y-maze data set is presented with outliers included. ROUT analysis at Q = 0.1% identified the highlighted data point as highly probable outliers, and the *p value was calculated on this basis. (C) Barnes maze probe test performance was not altered by *R. gnavus* colonization (D-E) nor was primary error or primary latency. (F) Total error was significantly reduced in *Rg*-mice with this group, making fewer errors overall. (G) Total latency remained constant across both groups. (H) NOR performance appeared to be greatly impaired in GF mice with animals unable to distinguish between novel and familiar objects, and this was not restored upon colonization with *R. gnavus*. (I-J) OF test score was similar across both experimental groups. Data are presented as mean ± S.E.M.

Defective adult hippocampal neurogenesis can impair learning and memory.^51,52^ To evaluate whether the modulation of granule cell development and synaptic plasticity in the adult hippocampus of *Rg*-mice could alter their cognitive functions, we performed a battery of behavioral tests (Y-maze, Barnes maze, novel object recognition (NOR) and open field (OF)). Spatial learning and working memory were assessed through the combination of Y-maze and Barnes maze behavioral tasks (Figure 10A-G). Figure 10A shows differences in the distribution pattern of Y-maze tasks between GF and *Rg*-mice. Statistical analysis including the removal of two outlier mice from the GF group (see the Materials and Methods) showed an improved Y-maze task performance upon colonization by 27% (p < 0.05). Improvement in performance was not a product of locomotor activity, as determined via Pearson’s correlation (Figure 10B). Such an observation did not manifest in the Barnes probe test (Figure 10C), although subtle indications of improvement were apparent throughout the learning phase (Figure 10D-G) with *Rg*-mice making significantly less errors across the 7 trials (p < 0.05) (Figure 10F).

In contrast to spatial working memory performance, recognition memory was not influenced by *R. gnavus*. Both GF and *Rg-*mice showed impaired recognition memory performance, with both groups being unable to distinguish between novel and familiar objects (Figure 10H). Furthermore, the OF task, a measure of locomotor activity, anxiety-related behavior, and exploration, did not indicate any differences between the two experimental groups (Figure 10I-J). Together, these data suggest that *R. gnavus* colonization specifically affects spatial working memory performance.

## Discussion

Emerging evidence suggests that the gut microbiota is a key regulator of normal homeostasis in the GI tract as well as in the central nervous system.^53,54^ Transplantation of fecal microbiota into GF mice demonstrated the role of the gut microbiota in gut-brain signaling, but there is little information on the role of specific gut symbionts in modulating brain function.^55^ In this work, we investigated the role of the mucin glycan forager *R. gnavus* ATCC 29149 in metabolism and brain function. Sialic acids and fucose residues found in the terminal location of mucin glycan chains can be released and/or metabolised by gut microbes inhabiting the intestinal mucus layer.^56^ Changes in mucin glycosylation have been reported to occur in the GI tract of GF mice up to 5–6 weeks after colonization with the gut microbiota derived from conventionally raised mice.^57^ Here, following two weeks of colonization with *R. gnavus* ATCC 29149, we observed a decrease in sialic acid from Muc2 and mix of mucins in the small intestine and the colon, with a greater decrease observed in the latter. A decrease in the abundance of fucosylated structures was also observed in the colonic mucins. These results are consistent with the ability of *R. gnavus* ATCC 29149 to hydrolyze fucosylated and sialylated mucin glycans.^13,58^

Although most studies to date have focused on Neu5Ac or Neu5Gc as the main sialic acid forms in mammals, more than 80 naturallyoccurring derivatives of sialic acid have been described harboring a different chemical group at the C-5 position of the sugar ring.^59^ Neu5Gc is a common form of sialic acid present in mammalian^60^ but absent in normal human tissue due to a mutation of the gene encoding the CMAH hydroxylase, which converts CMP-Neu5Ac into CMP-Neu5Gc.^61^ In this work, we showed that levels of Neu5Gc were higher in the serum as compared to cecum and brain, in line with studies in specificpathogen-free (SPF) mice, showing that Neu5Gc is absorbed by the intestinal epithelium and circulates into the serum.^62^ In addition, we could detect the presence of Neu5Ac and its modified acetylated forms, Neu5,7Ac2, Neu5,8Ac2, Neu5,GcAc, Neu5,9Ac2, and Neu5,7(8)9Ac3 in the brain, serum, and cecal content of GF and *Rg-*mice. The acetylated forms Neu5,9Ac2, Neu5,7Ac2, and Neu5,7(8)9Ac3 have been reported in the epithelial cells, goblet cells, and mucus layers of stomach, small intestine, and colon.^63^ These structures may be influenced by the gut microbiota and pathogens,^64^ although *O*-acetylation of sialic acid in the gut (Neu5,7Ac2 and Neu5Ac,9Ac2) is partially resistant to the action of bacterial sialidase.^65^ Here, we showed that colonization of mice with *R. gnavus* ATCC 29149 affected the distribution of sialic acid derivatives in the cecal contents and some areas of the mouse brain. The brain represents the organ with the highest level of sialic acids,^66^ with Neu5Ac being abundant in the membranes of neurons and glial cells.^67^ We found that Neu5Ac was the main sialic acid structure in the brain, either free or when bound to glycoproteins. Moreover, we found a significant decrease of Neu5,7Ac2 in the frontal cortex of *Rg*-mice, whereas Neu5,8Ac2 was under-represented in the glycoprotein portion of *Rg*-mice brain when compared to GF mice. In tissues and cell lines of different mammals, the widespread expression of acetylated forms of sialic acid is often accompanied by the presence of the acetylated modified structure Neu5,7(8),9Ac3, which is due to the spontaneous migration of the *O*-acetyl groups in the hydroxyl groups of the sialic acid chain^64,68^ consistent with the significant increase of this sialic acid derivative in the frontal cortex of *Rg*-mice as compared to GF mice. The associations between *R. gnavus* colonization and alteration in levels of *O*-acetylated sialic acid forms in the gut and the brain may indicate a potential route between mucin-derived sialic acid metabolism by gut bacteria and the brain, which warrants further investigation to determine causality.

In addition to sialic acid derivatives, untargeted metabolomic analyses identified several *R. gnavus*-derived metabolites that could be engaged in the communication between the gut and the brain. Interestingly, tryptamine was only detected in the cecum of *Rg*-mice, in agreement with the unique ability of *R. gnavus* ATCC 29149 to convert tryptophan into tryptamine by the action of the tryptophan decarboxylase enzyme,^69^ a rare enzymatic activity among bacteria.^70^ In the gut, tryptamine is known to induce the release of the neurotransmitter 5-hydroxytryptamine (5-HT, serotonin) by enterochromaffin cells and modulate the circulating levels.^69,71^ *Rg*-mice also showed a significant increase of indolacetate and indolacetylglycine in the cecal content and serum as compared to GF mice. Previous studies reported that GF rodents exhibit elevated tryptophan levels and reduced indole derivatives in serum.^72,73^ Indole-derived metabolites can influence the proliferation of neural progenitor cells in the hippocampus through the aryl hydrocarbon receptor,^72^ and some of them have shown anti-inflammatory effects on the brain.^74^ TMAO, an important metabolite whose production depends on gut microbiota metabolism, was found to be increased in the serum of *Rg*-mice. The gut microbiota can produce trimethylamine (TMA) from carnitine, choline, and choline containing compounds from the diet.^75^ TMA is further oxidised in TMAO in the liver, kidney, and other tissues where it is linked with inflammatory events.^76^ Correlations have been reported between altered levels of TMAO and neurological disorders, such as Parkinson’s disease^77^ or atherosclerosis and cardiovascular diseases.^78^ Recently, TMAO was shown to have protective effects on BBB integrity, while its precursor TMA impaired BBB integrity.^79^ In addition, *R. gnavus* was found to be increased in patients suffering from cardiovascular diseases and atherosclerosis^76^ with high levels of TMAO^80^ and was recently proposed as a candidate producing this proinflammatory metabolite,^81^ although direct evidence remains to be demonstrated. The increased production of tryptamine, indole, and choline metabolites in *Rg*-mice may therefore provide a mechanistic link underpinning the regulation of brain function and behavior in these mice as further discussed below.

To investigate the role of *R. gnavus* in the gut-brain axis, we first determined the effects of *R. gnavus* ATCC 29149 monocolonization on PSA-NCAM^+^ cell lineage in the hippocampus. Although the levels of cell proliferation, as determined by BrdU incorporation, were not significantly different between the two experimental mouse groups, a significant decrease in the number of PSA-NCAM^+^ granule cells (amplifying and immature cells) was observed in the neurogenic site of *Rg*-mice as compared to GF mice, indicating a regulation of the differentiation and amplification processes of the new-born cells in the neurogenic niche. In contrast, the PSA-NCAM^+^ cell lineage in control GF mice showed an increased survival rate of early progenitor cells with few granule cells holding a different maturation morphology. It is of note that GF mice were colonized with *R. gnavus* at ~10 weeks old, confirming the importance of an appropriate time window in mediating gut microbiota effects since colonization of GF mice with gut microbiota at 3 weeks old (postweaning stage) was previously shown not to influence neurogenesis.^82^ In the hippocampal SGZ, *ST8sia2* is the main polysialyltransferase expressed in immature neurons and altered levels in *ST8sia2* expression have been linked with schizophrenia, as determined by analysis of postmortem brain of schizophrenic patients.^83,84^ In addition, ST8sia2-deficient mouse models displayed a decreased in NCAM polysialylation and a disrupted phenotype of granule cells, reflecting an impairment in the spatial working memory, both features of schizophrenia-like phenotype.^24,25^ Here, we showed an upregulation of *ST8sia2* as well as *Ncam1* in *Rg-*mice, suggesting a modulation of NCAM polysialylation, which will impact synaptic plasticity and memory consolidation. Furthermore, PSA-NCAM is strongly affected by the expression of the CREB signaling pathway, a known neurogenic factor regulating genes related to neuronal differentiation, synaptic plasticity, learning, and memory.^85,86^ *Rg*-mice showed a significant upregulation of *Creb1*, as compared to GF mice, suggesting a higher survival rate of immature granule cells in the DG and integration of new neurons in the hippocampal circuits.^86,87^ These results are in line with previous *in vivo* mouse studies where CREB has been found to be associated with newborn immature neurons and amplified neurite outgrowth and branching.^86^ Therefore, the impact of *R. gnavus* monocolonization on hippocampal neurogenesis seems to act on shaping the morphology of the granule cells, which contrasts from GF mice where the higher hippocampal cell expansion is composed of less branched and differentiated granule cells.^88^

To our knowledge, no studies investigating the role of gut microbes in the gut-brain axis have investigated the role of PSA-NCAM and polysialylation in GF and gnotobiotic mice. However, in agreement with our findings, monocolonization of GF mice with tryptophan-metabolizing gut bacteria was shown to increase hippocampal neurogenesis and survival of new-born neurons,^72^ which may suggest a role in *R. gnavus* tryptophan-derived metabolites, such as tryptamine, in the regulation of the new-born/immature granule cell development and regulation. On the other hand, reconstitution of antibiotic-treated adult mice with SPF fecal transplant was not sufficient to improve impaired hippocampal neurogenesis,^29^ whereas voluntary exercise enhanced it, stressing the complexity of the processes linking gut microbiota and brain.^89^

Another potential mechanism by which gut microbes have been shown to regulate the expansion of new-born cells is through the phagocytic activity of microglia.^90^ GF mice display global defects in microglia with altered cell proportions and an immature phenotype, leading to impaired innate immune responses and recolonization with a complex microbiota partially restoring microglia features through the regulation of microglia maturation by microbiota-derived bacterial fermentation products.^91^ Here, we showed that in the SGZ of *Rg*-mice, an higher number of microglia cells (Iba1^+^) were observed to be in close proximity to granule cells (PSA-NCAM^+^). During inflammatory insult, increased apoptosis of neural progenitor cells is coupled to increased phagocytosis by SGZ microglia, suggesting that the removal of apoptotic progenitors by microglia is required to maintain neurogenesis.^92,93^ The phagocytosis of apoptotic neurons depends on receptors such as TREM2 (triggering receptor expressed on myeloid cells 2), becoming activated to engulf dying cells in the vicinity.^92,93^ Here, in comparison to GF mice, *Rg*-mice displayed a significant decrease of the expression levels of *Gfap*, identified as a marker of proliferating cells/neuroblast in the developing population of the DG^94,95^ and a significant upregulation of *Trem2*, indicating a contribution of microglia in modulating the expansion of granule cells. These results are in line with previous studies showing that microglia cells in GF mice are not reactive and therefore unable to define the synaptic pruning of granule cells in the cerebellum region by phagocytic activity, a situation that could be reverted by colonization with Bifidobacterium species, showing an increase of synaptic plasticity and microglia pruning.^89^ Nevertheless, although the impact of *R. gnavus* on the proliferation of granule cells resulted in an increase of unchallenged microglia activity, the analysis of microglia morphology did not show significant differences between *Rg*-mice and GF mice, most likely due to the requirement of a complex microbial community in an early developmental stage for the maturation of the heterogenous microglia population and immune functions.^96,97^

The gut microbiota and associated metabolites are known to influence the BBB permeability,^98,99^ with GF mice showing alterations in tight-junction proteins as compared to SPF mice.^100^ Interestingly, neurogenesis seems to be accompanied by a temporary increased permeability of the BBB so to facilitate and accommodate the nutrient supply needed for the proliferation state of the cells.^101^ Then Upregulation of tight junctions occurs when new neurons are formed and further differentiated.^102^ Previous work showed that increased BBB permeability of GF mice during adulthood may be partly due to disorganized tight junctions and low expression of occludin and claudin-5.^101^ This could be reverted to levels equivalent to that of SPF adult mice following fecal transplantation from SPF mice or monocolonization of GF mice with bacterial strains that produce SCFAs, *Clostridium tyrobutyricumm* or *Bacteroides thetaiotaomicron*.^100^ Here, *Rg*-mice showed a significant decrease in the expression levels of occludin and claudin-1, whereas ZO-1 showed an higher expression as compared to GF mice, suggesting changes in BBB permeability that may be related to the observed effects on neurogenesis in these mice. Although no significant changes in SCFA levels were detected between GF and *Rg*-mice, these effects may be mediated by the increased levels of TMAO in the serum of *Rg*-mice, as recent studies showed that physiologically relevant concentrations of TMAO have positive effects upon BBB integrity.^79^

We next determined how these molecular and morphological changes in the neurogenesis and/or development of new-born neurons in the hippocampus of *Rg*-mice may influence the behavior of these mice as compared to GF mice. *Rg*-mice was found to perform better in the Y-maze test, indicating an increase in spatial memory performance as compared to GF mice, while anxiety, locomotor activity, and object recognition memory did not show differences between *Rg*-mice and GF mice. Previous studies in rodents showed that members of the gut microbiota or probiotic strains are able to modulate host behavior in a strain-specific manner. For example, colonization of adult GF mice with four “infant type” Bifidobacterium species from day 1 after birth until weaning followed by weekly administration of the probiotic rescued the anxiolytic behavior of GF mice and improved the locomotor activity and recognition memory tasks of these animals.^103^ In diabetic rats, the administration of probiotics *Lactobacillus acidophilus, Bifidobacterium lactis*, and *Lactobacillus fermentum* was able to re-establish synaptic activity in the hippocampus and improve spatial learning tasks through upregulation of synaptic plasticity markers.^104^ Likewise, *Akkermansia muciniphila* administration enhanced spatial memory in mice fed on a high fat diet^105^ and prevented age-related memory dysfunctions in rats.^106^ It is likely that gut symbionts contribute to these behavioral changes through different mechanisms depending on their metabolic capacity and niche in the gut. Here, the improvement of spatial learning tasks in *Rg*-mice appeared to be linked to the successful development and survival of granule cells in the hippocampal network of these mice, a process that has been shown to be critical to spatial learning related memory.^107,108^ In addition, the upregulated expression of *ST8sia2* and *Ncam1* in *Rg*-mice is in line with the importance of PSA-NCAM for the development and adult neuroplasticity.^109,110^ These effects may be mediated by the increased levels of tryptophan and indoles in *Rg*-mice, as recent studies showed that microbial-derived indoles have neuroprotective properties, can cross the BBB, and rescue neurogenesis in GF mice.^70^

Collectively, these data provide first insights into how *R. gnavus* may influence brain regulation and and spatial working memory behavior through the production of metabolites. Despite the limitations associated with the use of gnotobiotic mouse models in terms of gut development and physiology, our findings showed that monocolonization with *R. gnavus* affected the differentiation of granule cell lineage through increased activity of phagocytic microglia and synaptic plasticity. Further work is required to tease out which *R. gnavus* specific metabolites mediate these effects.

## Materials and methods

### Mouse monocolonization

Germ-free C57BL/6 mice were bred in house at the Disease Modelling Unit (DMU, University of East Anglia, Norwich). All experiments were performed in accordance with regulations under the UK Animals (Scientific procedures) Act 1986. All efforts were made to minimize animal suffering during the experiments. Germ-free C57BL/6 female mice (n = 10; age, ~8–10 weeks old) were housed 5 per cage in two different isolators. The 10 mice were fed with an increasing amount of custom-made sialic acid-free diet over a period of 2 weeks to ensure adaptation and washing out of any residual sialic acid from the normal diet. Then, two studies were carried out where 5 mice from one isolator received 1 dose of *R. gnavus* ATCC 29149, whereas the other 5 mice remained germ-free and were used as control. *R. gnavus* ATCC 29149 was grown in an anaerobic cabinet in Brain Heart Infusion broth supplemented with yeast extract and hemin (BHI-YH) as previously described.^11^ GF mice were gavaged with 1 × 10^8^ CFU in 100 µl of PBS (phosphate buffered saline solution), whereas 100 µL at 2.5 × 10^8^ CFU was used in the group receiving the 5’-bromo-2- deoxyuridine (BrDU Merk 19–160) treatment. Fecal samples were collected just before gavage (D0), 7 days after gavage (D7), and at D14. The mice were culled by Schedule-1 at D14, and serum, cecal contents, and brain were collected for sialic acid quantification analysis. Mucus was scraped from small intestine and colon for mucin glycosylation analyses. The same experimental design was applied for the neurogenesis study with mice treated with BrdU. Mice were culled, and brains were dissected out and fixed overnight in 4% paraformaldehyde (PFA, pH 7.4 Merk 158,127) in phosphate-buffered saline solution (PBS). The brains were then dehydrated stepwise into absolute ethanol and stored in the same at 4°C until use. For the behavioral study, germ-free C57BL/6 female mice (n = 16; age, ~8–10 weeks old) were housed in two different isolators and gavage with one dose of *R.* *gnavus* ATCC 29149 1 × 10^8^ CFU in 100 µl of PBS as above (n = 8 *R. gnavus* treated, n = 8 GF) and subjected to the behavioral battery tests for 5 days.

### Quantification of R. gnavus by quantitative qPCR

DNA was extracted from fecal samples using the Fast DNA™ SPIN kit for soil DNA extraction (MP Biomedicals, USA) with the following modifications. Briefly, the fecal pellets were resuspended in 978 μL of sodium phosphate buffer before being incubated at 4°C for 1 h following addition of 122 μL of lysis solution MT buffer. The suspensions were then transferred into the lysing tubes and homogenized in a FastPrep® Instrument (MP Biomedicals) 3 times for 40 sec at a speed of 6.0 m/s with a 5-min interval on ice between each bead-beating step. The protocol was then followed as recommended by the supplier. DNA quality and quantity were determined using the Qubit dsDNA HS assay on a Qubit® 2.0 fluorometer (ThermoFisher Scientific, UK). Dilutions at 10 ng/μL were prepared in RNA-free water, and then the DNA was diluted further in 10 μL of RNA-free water containing 5 μg/mL salmon sperm DNA to obtain a 1 ng/μL dilution used as a template for the qPCR. The qPCR was carried out in an Applied Biosystems 7500 Real-Time PCR system (Life Technologies Ltd). Two pairs of primers targeting the 16S gene of all bacteria (universal primer) and the 16S gene of *R. gnavus* strains (Rg_16S_5 F/Rg_16S_5 R) (**Table S4**) were used. Each qPCR reaction (10 μL) was then carried out in triplicates with 2 μL of DNA matrix at 1 ng/μL and 0.5 mM of each primer, using the QuantiFast SYBR Green PCR kit (204,054 Qiagen) according to supplier’s instructions (except for the combined annealing/extension step that was extended to 35 sec).

Data were analyzed using the standard curves prepared in triplicates for each pair of primers using dilutions ranging from 10^7^ copies to 10^2^ copies of 16S PCR products. The standard curves showed a linear relationship of log input copy number vs. the threshold cycle (C_T_), with acceptable values for the slopes and the regression coefficients (R^2^). The dissociation curves were also constructed to verify the specificity of the amplicons.

### RNA isolation and qRT-PCR

Briefly, total RNA was isolated from the brain samples using the Qiazol reagent (Qiagen, UK). One μg of total RNA was treated with DNase I (Invitrogen, UK) and used for cDNA synthesis using Invitrogen Oligo (dT) primers and M-MMLV reverse transcriptase. Quantitative real-time PCR (qRT-PCR) reactions were performed using SYBR green detection technology on the Roche light cycler 480 (Roche Life Science, UK). Results are expressed as relative quantity scaled to the average across all samples per target gene and normalized to the reference gene glyceraldehyde 3-phosphate dehydrogenase (*Gapdh*), which was identified as the optimal housekeeping selection using the software RefFinder.^111^ Primer sequences are given in **Table S5.**

### Behavioral assessment

All behavioral tests were performed when mice reached 7–12 weeks of age. Tests were performed as follows: Y maze and OF were completed on day one immediately after removal from germ-free environment. NOR was completed on day 2, and Barnes maze was conducted from days 3 to 5. A visual placing test was performed on each animal on the first day of testing, to ensure that animals were not visually impaired. Mazes were cleaned in between each trial with 70% ethanol.

The Y-maze spontaneous alternation test, a measure of spatial working memory, was conducted as previously described.^107^ Briefly, mice were placed into the Y-maze apparatus and allowed to explore freely for 8 min. Mice were tracked using software that determined zone transitioning and locomotor activity (Smart 3.0 tracking software, Panlab, Kent, UK). The following formula was used to determine spontaneous alternation: spontaneous alternation = (number of alternations/total arm entries − 2) × 100.

Spatial learning and memory were evaluated through the Barnes Maze as described previously but with slight modifications.^107,108^ Briefly, the maze was brightly illuminated (800 lux lighting), and the animal was placed onto the circular platform (92 cm diameter) and was trained to find the designated escape box among the 20 evenly distributed holes located around the circumference using visual cues (4 simple shapes) placed around the periphery. The experiment was conducted over a 3-day period, with training consisting of 7 trials on days 1 and 2. On day 3, a probe test was conducted, the maze was rotated 90°, the escape box was removed, and mice were placed in the center of the maze in which they were free to navigate for 90 sec. The percentage time in the correct quadrant was determined using Ethovision software (UK).

The OP task, a measure of anxiety, locomotor activity, and exploration, was performed as described. Briefly, mice were placed into an empty gray 50 × 50 × 50 cm apparatus illuminated with low lux 100 lux lighting and allowed to move freely for 10 min. Mice were tracked using ethovision software that determined the travel distance and time spent in the center of the maze. Recognition memory was assessed using the novel object recognition (NOR) task and was performed as described previously^112^ with slight modifications. The OF task served as the day 1 habituation for the NOR. On day 2, mice were placed into the same experimental area in the presence of two identical objects for 15 min, after which they were returned to their respective cages and an intertrial interval of 1 h was observed. One familiar object was replaced with a novel object. Mice were placed back within the testing area for a final 10 min. Videos were analyzed using ethovision software for a 5-min period, after which if the total object exploration time (nose within object zone) failed to reach an accumulative 10 sec, analysis continued until the 10 sec was met. All animals achieved at least 10 sec of interaction. The following formula was used to calculate discrimination index: **DI = (TN − TF)/(TN + TF)**, where TN is the time spent in exploring the novel object and TF is the time spent in exploring the familiar object.

### Sialic acid extraction

All steps for the extraction of sialic acid were performed at 4°C to minimize enzymatic hydrolysis. For extraction of sialic acids from the mouse cecal content, the protocol was as previously described.^113^ Briefly, approximately 200 mg of flash-frozen cecal contents were resuspended in 400 µL of dH_2_O. Samples were vortexed for 30 min and centrifuged for 30 min at 14,000 × g at 4°C. The supernatant was stored at 4°C, and the pellet resuspended in 400 µL of dH_2_O. This process was repeated until approximately 1 mL of pooled supernatant was obtained. Each sample (700 µL) was filtered through a Pall 1 K MWCO filter for 9 h at 7,000 × g. The filtrate was collected and stored at 4°C overnight until labeling and HPLC analysis.

For extraction of sialic acids from brain tissues, the protocol was as previously published ^114^ with modifications. The snap frozen brain regions corresponding to the frontal cortex and midbrain (about 100 mg) were homogenized with 500 µL of chloroform:methanol (C:M 2:1, v/v) using a FastPrep homogenizer (1x3 cycle at 6.0 Hz, 40 sec). Then, another 500 µL of C:M (2:1, v/v) was added to the suspension and mixed for 20 min at 4°C at a 1000 x g speed using a vortex. The homogenate was centrifuged for 20 min at 10,000 g at 4°C. The supernatant containing the glycolipids and gangliosides was collected and kept at 4°C until further analysis. The pellet was incubated with 1 mL of C:M (1:2, v/v), followed by two successive treatments with C:M (2:1, v/v) and C:M (1:2, v/v) and centrifuged as above. The resulting pellet (insoluble glycoproteins) was resuspended with 500 µL of 10 mM Tris-HCl pH 7.4 buffer and centrifuged for 20 min at 10,000 g at 4°C. The supernatant was precipitated by incubation with a 70% ethanol final concentration at −20°C overnight and centrifuged for 20 min at 12,000 g and 4°C. The supernatant containing the cytosolic free sialic acids was dried in a speedvac for 3 h and stored at −20°C. The pellet containing 200 µL of Tris-HCl soluble glycoproteins (*N*- and *O*-glycoproteins) was stored at 4°C until further analysis. The fraction of the brain containing insoluble glycoproteins was precipitated following incubation with a 70% ethanol final concentration at −20°C overnight and centrifugation for 20 min at 12,000 x g and 4°C. The supernatant was carefully discarded without disturbing the pellet. Once dried, the pellet was treated for mild acidic hydrolysis with 4 N acetic acid, followed by labeling and HPLC analysis.

For extraction of sialic acid from the serum, blood (100–200 µL) was collected from intracardial puncture of mice following schedule-1 culling and left at room temperature for approx. 30 min to allow clotting. The samples were centrifuged at 3,000 rpm for 10 min at 4°C, and the serum (supernatant) was collected and stored at −80°C until analysis. For sialic acid derivative quantification, an initial dilution of 1/100 of serum with MilliQ water in a final volume of 100 µL was used for mild acidic hydrolysis with 4 N acetic acid.

### HPLC analysis of sialic acid derivatives

Sialic acids were first fluorescently labeled by 1,2-diamino-4,5-methylenedioxybenzene dihydrochloride (DMB FD21461, Carbosynth). Briefly, samples were first treated through a mild acidic hydrolysis in 100 µL of 2 N acetic acid for 3 h at 80°C, dried and then resuspended in 50 µL of MilliQ water and 50 µL of DMB reaction buffer as described below, and incubated in a heating block for 2.5 h at 55°C in the dark. Briefly, for ten samples to be labeled, a solution containing 1.74 mg of DMB, 324.6 µL of MilliQ water, 88.6 µL of glacial acetic acid, 58.2 µL of 2-mercaptoethanol, and 79.3 µL of sodium hydrosulfite was prepared. The samples were then centrifuged for 1 min and filtered through a 0.45 µm polytetrafluoroethylene (PTFE) filter membrane (6784–2504 GE Healthcare, Like Sciences) into a screw cap glass vial and stored at −20°C until further analysis or directly analyzed by HPLC. For sialic acid quantification, a calibration curve was prepared using 6 concentrations of Neu5Ac ranging from 5 to 0.02 ng/µL (from a stock concentration of 0.5 mg/mL) and labeled with DMB as described above. DMB-labeled samples were analyzed using a Luna 5 µm C-18 (2) LC column 250 × 4.6 mm (Phenomenex Manufactures) on an HPLC (Prominence Shimadzu). Samples (10 µL) were injected to the column at a flow rate of 1.0 mL/min at 30°C. The mobile phase consisted of water (A), 14% acetonitrile (ACN) (B), and 50% methanol (C). The fluorescence was detected at 373 nm (excitation wavelength) and 448 nm (emission wavelength). Sialic acid derivatives underwent a starting equilibration step with the solvents for 10 min and then separated on a gradient from 82:4:14 A/B/C to 75:11:14 over 40 min, followed by a wash step with 30:70:0 A/B/C for 10 min. Standard regression and sample quantitation were calculated using Microsoft Excel.

### MALDI-ToF MS mucin glycosylation analysis

Mucus scraped from small intestine and colon of mice was pooled by GI location and mice group and resuspended at least 5–8 times the volume of sample with 6 M guanidine chloride (GuCl) buffer (prepared in MilliQ water) containing protease inhibitors (7.95 mM ethylenediaminetetraacetic acid (EDTA 60–00-4 Merk), 12.25 mM benzamidine (B6506 Merk), 6.25 mM *N*-ethylmaleimide (E3876 Merk), 1.25 mM phenylmethylsulfonyl fluoride (PMSF 36978 ThermoFisher), 3.75 mM sodium azide (S2002 Merk), and 0.1 mg/mL soybean inhibitor (17,075,029 ThermoFisher). The samples were left to solubilize for 2 days at 4°C on a rotary wheel at 112 x g. Next, the samples were centrifuged at 16,000 x g using an Eppendorf F34-6-38 rotor (fixed angle rotor) at 4°C for 30 min. The pellet contained the Muc2 protein fractions, while the supernatant contained mix mucins as previously reported.^115^ The pellet was reduced with dithiothreitol (DTT 3483–12-3 Merk) at 10 mM in GuCl 6 M for 4 h at 45°C and alkylated with 25 mM iodoacetamide (144–48-9 Merk) overnight. After centrifugation, the supernatant was dialyzed against 50 mM NH₄HCO₃ in a dialysis tubing (MWCO 12–14 kDa) for 2 days, by changing the buffer twice a day. Next, the samples were freeze-dried and stored at 4°C until use. The supernatants containing soluble mix mucins were diluted in 4 M GuCl in PBS, and the density was adjusted with cesium chloride (CsCl 7647–17-8 Merk) to 1.4 g/mL.^−1^ Supernatants were subjected to ultracentrifugation (Beckman, Brea, US) at 54,000 rpm for 72 h at 20°C. Fractions of 1 mL were collected and weighed. Fractions between 1.35 and 1.45 g/mL^−1^ containing the purified mix mucins were pooled and dialyzed against 50 mM NH₄HCO₃.

Glycans were released from ~1 mg of mucin by β-elimination in 0.5 ml of 0.5 M NaBH_4_ in 50 mM NaOH. The reaction was incubated at 45°C for 16 h and quenched by the addition of 50 μL of acetic acid. The samples were desalted on an in-house packed Dowex H^+^ column, borates were removed by coevaporation with methanol, and the samples were freeze-dried. The glycans were permethylated as described before^116^ and analyzed by MALDI-ToF MS on a Bruker Autoflex (Bruker Daltonics, Bremen, Germany).

### Metabolomics

The cecal content (100–200 mg) and serum (50–60 µL) from *R. gnavus* ATCC 29149 monocolonized mice (n = 5) and germ-free mice (n = 5) were processed and analyzed by Metabolon, Inc, USA. A total of 633 compounds of known identity (named biochemicals) were detected in cecal contents, and 618 detected the serum (see the Supplementary Information). For the metabolites identification, Metabolon used a library based on authenticated standards that contains the retention time/index (RI), the mass to charge ratio (m/z), and chromatographic data (including MS/MS spectral data) on all molecules present in the library. Furthermore, the biochemical identifications were based on three criteria: retention index within a narrow RI window of the proposed identification, accurate mass match to the library ± 10 ppm, and the MS/MS forward and reverse scores between the experimental data and authentic standards.

### 5-Bromo-2′-deoxyuridine (BrdU) in vivo treatment

5-Bromo-2′-deoxyuridine (BrdU) was dissolved in PBS (20 mg/mL) at 56°C by constant vortexing, and the solution was filter-sterilized through a 0.2 µm filter. To label proliferating cells, mice were given a 100 µL intraperitoneal (i.p.) injection corresponding to a 300 mg/kg bodyweight dose as previously described.^117^ After 24 h, the mice were culled by Schedule-1 and colon and brain were harvested and fixed overnight in paraformaldehyde (PFA) at 4°C. The following day, the samples were washed with PFA and processed for immunohistochemistry analyses.

### Preparation and immunolabeling of brain sections

On the day of use, brains that have been previously fixed in 4% PFA and dehydrated into ethanol were rehydrated in descending (90%, 70%, 50%, and 30% and PBS; 1 h each step). The brains were then embedded in 3% agar, and serial 60 µm slices were cut using a vibratome (Leica, VT1200S). The floating brain sections were then transferred into wells of a 48-well plate (Corning, UK) filled with PBS. For immunolabeling, free floating sections from the region of interest (Bregma coordinates from −1.22 mm to −2.46 mm, encompassing hippocampus and lateral subventricular zone) were selected with the aid of an atlas (Paxinos and Franklin’s the Mouse Brain in Stereotaxic Coordinates, Compact 5th Edition). Sections were then subjected to antigen retrieval by a 15 min treatment at 70°C with 10 mM citrate buffer, pH 6.0/0.05% Tween 20, in a water bath. For BrdU staining, 2 M HCl at 37°C was used for antigen retrieval, followed by two 10-min washes in PBS. The sections were then blocked for 2 h with a PBS solution containing 20% normal goat serum (NGS 10098792 Gibco) and 1% Triton X100 and then incubated overnight at 4°C with primary antibodies (**Table S6**) in a solution containing 0.2% NGS and 0.1% Triton X100. The sections were then washed five times by incubation for 1 h each at room temperature in 0.2% NGS and 0.1% Triton X100 and then incubated overnight at 4°C with the relevant secondary antibodies diluted in the buffer used for the primary antibodies (**Table S6**). Following washing in PBS (6 times, 30 min incubation each), the sections were washed, mounted, and cover-slipped with mounting medium (Antifade Mounting Medium- H100 VECTASHIELD).

### Image acquisition

Imaging was performed using a LSM 880 confocal laser scanning microscope with an Airyscan (ZEISS). The images were acquired using 10× and 20× objectives. For morphology analyses, confocal microscopy was carried out using the following settings: 60-µm Z-stack at 13.5-µm intervals (12–15 slices) with a 20× objective and a 708.49 × 708.49 imaged area (2048 x 2048 pixels). Between 4 and 7 slices per mouse were counted for all mice in the experimental groups under study, and descriptive analyses were conducted for each marker within each section or region in paired animals. The slices were used to perform a quantitative or qualitative analysis. Microglia, proliferating cells, and granule cells were labeled using specific antibodies (**Table S6**). Unless otherwise stated, photomicrographs were stacked and split using ImageJ plugins, in order to obtain maximum intensity projections of all channels, and processed for further quantification.

### Microglia cell analysis

To investigate the ramifications and cell complexity of microglia, plugins AnalyzeSkeleton (2D/3D) and FracLac were applied as previously described. ^118^ The skeleton analysis plugin, which tags skeletal features relevant to microglia ramification, was applied, and the resulting slab voxels (orange, process length) and end point (blue) were measured. Next, the plugin FracLac was used to determine fractal dimension (complexity), lacunarity (heterogeneity), density (size), and span ratio (elongation) of microglia cells. Eight microglia cells were randomly selected from the image also used for the skeleton analysis. Each image cell was first converted to binary and then to outlines using ImageJ. When the FracLac plugin was applied to the outline image, cell morphology parameters such as fractal dimension, lacunarity, density, span ratio, and circularity were obtained. To analyze the association of microglia with granule neurons expressing PSA-NCAM, Iba-1-positive cells were counted in 12–14 stack sections of GF mice (15 brain slides) and *Rg*-mice (17 brain slides) randomly selected from the total GF (n = 5) and *Rg*-mice (n = 5) brain samples. The first and the last stack sections were excluded from the counting.

### Analysis of PSA-NCAM immunoreactive cells

The dendrites length of PSA-NCAM granule neurons was quantified using plugin NeuronJ in ImageJ as previously described. ^119^ Photomicrographs of the hippocampus from both hemispheres were stacked using ImageJ plugins, in order to maximize intensity projections. Neurites extending from the granule cells lying in the subgranular zone of the dentate gyrus (DG) were manually traced and labeled. Only the primary branches were counted in the analysis. Based on the tracings, NeuronJ was used to measure the sum (total length), the mean, the standard deviation, and the minimum and maximum tracing length, as well as the mean, standard deviation, and minimum and maximum of the image values found along the selected tracings.

### Statistics

All statistical analyses were conducted using the R version 4.1.0. For the glycan analysis from intestinal mucins, at least three technical replicates were performed for each fraction and t-tests were performed to compare each fraction between GF and *Rg-*mice.

For the analysis of sialic acid derivatives between mouse types, sialic acid derivatives were modeled for each area separately. In each area, log-concentration was modeled using a linear mixed model with a random intercept of mouse and the interaction of the derivative and mouse group as fixed effects. The random intercept was added following visual inspection of the data and observation of intraclass correlation of different acids within mice. P-values for the effect of *R. gnavus* ATCC 29149 monocolonization on sialic acid patterns were calculated by comparing this full model with a model without any terms corresponding to the mouse group.

For the comparison of metabolites, each metabolite concentration was initially scaled and missing values were imputed as the smallest detected. Metabolite values were initially log-transformed prior to analysis. Average log-concentrations were calculated for each mouse across all metabolites in the serum and cecal contents separately and were subtracted from each mouse value to adjust for mouse-specific effects. Unpaired t-tests were then conducted for each metabolite in the cecal contents and serum separately. Metabolites were classified according to superpathways and subpathways. The proportion of metabolites that were significantly different between groups was compared across superpathways using the hypergeometric test. Adjusted p-values (q-values) for the effect of *R. gnavus* on individual metabolites were calculated using the method of Benjamini and Hochberg, individually for serum and cecal contents.

Cell counts (BrdU-positive cells and PSA-NCAM-positive cells) were analyzed using negative binomial linear mixed models, using the glmmTMB package (version 1.0.2.1). The main effects of the cell type and mouse group (GF mice vs *Rg*-mice) as well as their interaction were included as fixed effects, along with the bregma position and orientation. Random effects of individual mouse and slide nested within mouse were added separately for each cell type.

Group margins (averages) with 95% confidence intervals and associated marginal effects were calculated from all models using the emmeans package (version 1.5.5–1).

Branch-point lengths and end points of microglia were compared using linear mixed models, with the mouse group and brain area as main effects and mouse as a random effect, using the lme4 package (version 1.1–26) for R (version 4.1.0).

For the behavioral tests, all data are presented as mean ± S.E.M. Data analysis was performed in GraphPad Prism version 8 (GraphPad Software, CA, USA). After identifying outliers using the ROUT method, data were checked for normality/equal variances. Comparisons among groups were performed on normally distributed data using Student’s t-tests. Pearson’s correlation was used to assess the association of the travel distance and movement speed on corresponding behavioral test performance. P values of less than 0.05 were considered statistically significant.

## Supplementary Material

Supplemental Material

## Data Availability

The authors confirm that the data supporting the findings of this study are available within the article and its supplementary materials.
