# Supplementary material for: The role of the mucin-glycan foraging Ruminococcus gnavus in the communication between the gut and the brain
Source: Gut Microbes. 2022 May 17;14(1):2073784. doi: 10.1080/19490976.2022.2073784 (PMC9122312; doi:10.1080/19490976.2022.2073784)
Supplement: Supplemental Material [file KGMI_A_2073784_SM5980.zip › Revised Coletto et al Supplementary Information.docx]

Supplementary Information

**Table S1.** **Diet formulation used in the study and vitamin mix composition table**


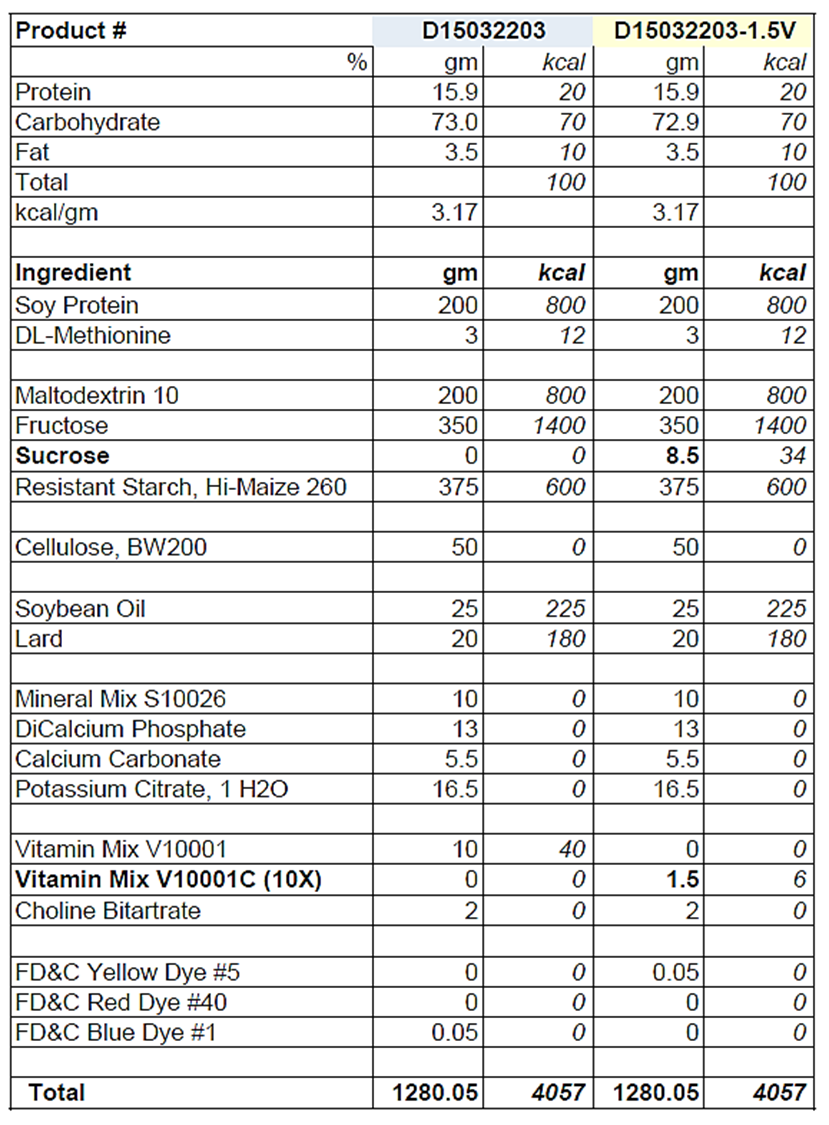


D15032203 represents the custom-made sialic acid-free diet used in the study.


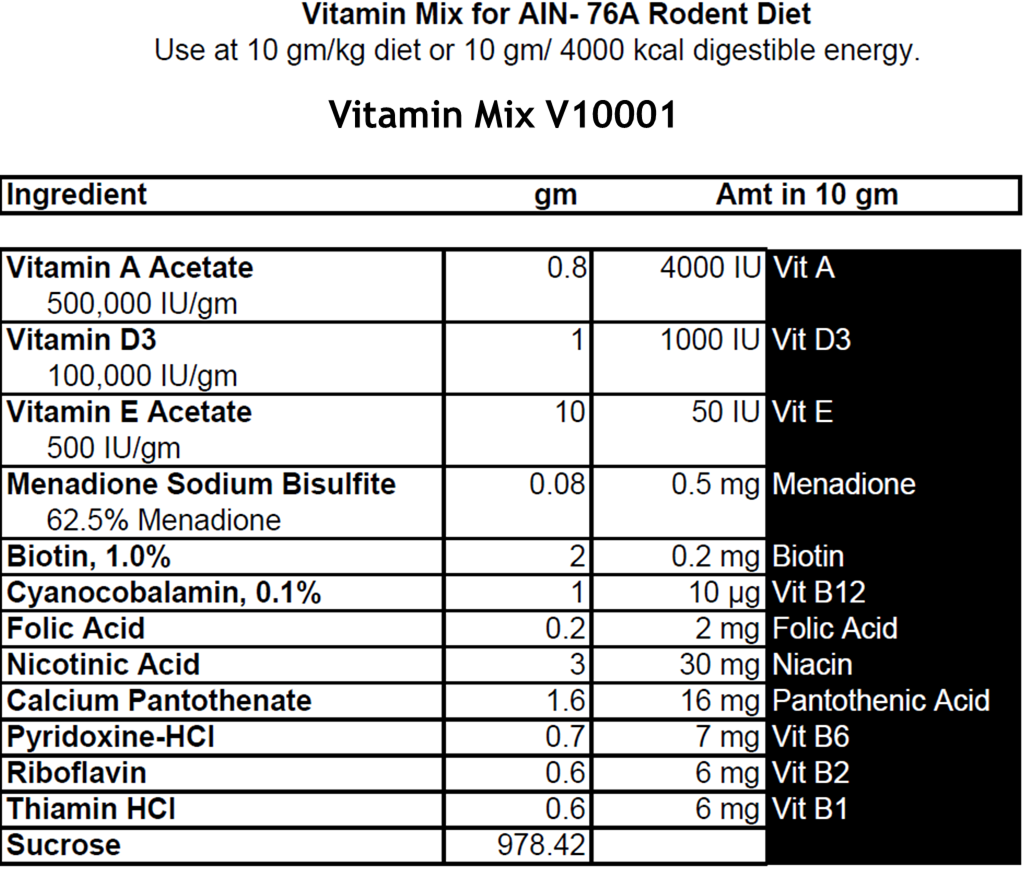


Vitamin Mix V10001 composition added to the diet.

**Table S2**. **Estimated average concentrations of sialic acid derivatives in GF-mice and *Rg*-mice**


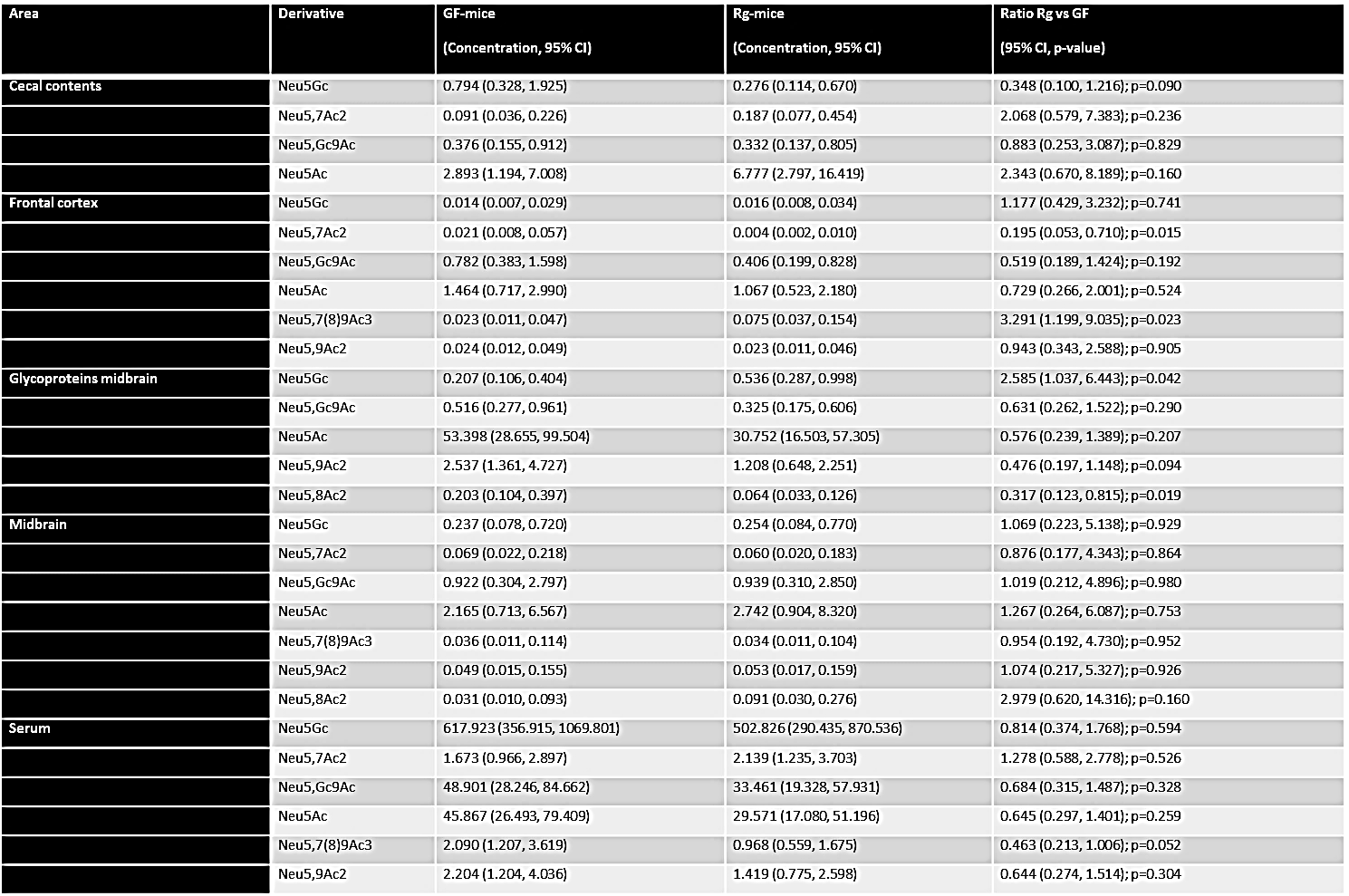


The analysis provides estimates for the effect of *R. gnavus* mono-colonization on each derivative (expressed as a ratio of concentrations) with 95% confidence intervals and p-values corresponding to the null hypothesis of ratio=1.

**Table S3. Statistical analysis of metabolites between GF and *Rg*-mice**


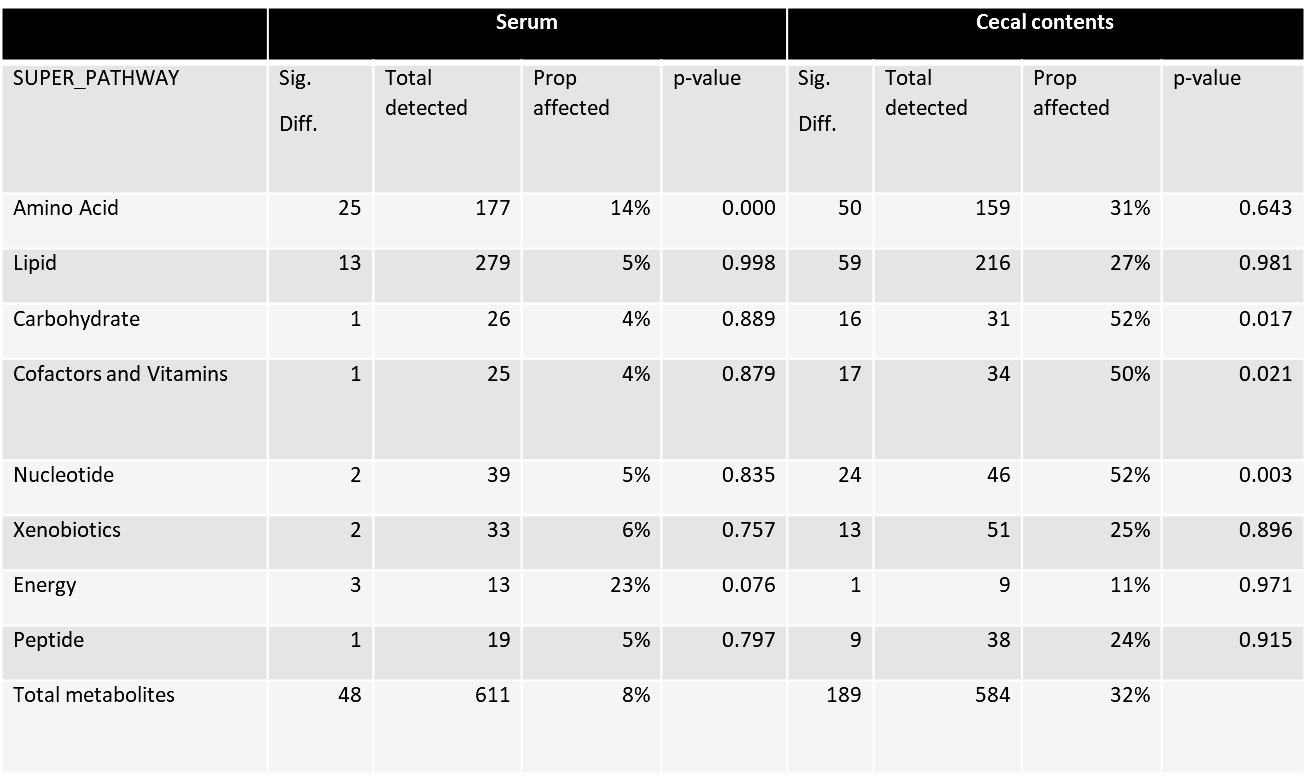


In the table, are shown the number of metabolites that are significantly different between groups (at p<0.05) out of the total number for each super-pathway. P-values correspond to the hypergeometric test comparing the proportion of tests that are statistically significant in each pathway to the number that are significant overall in each area (serum or cecum).

**Table S4. List of primers used for monitoring colonization of *R. gnavus* ATCC 29149 in gnotobiotic mice**


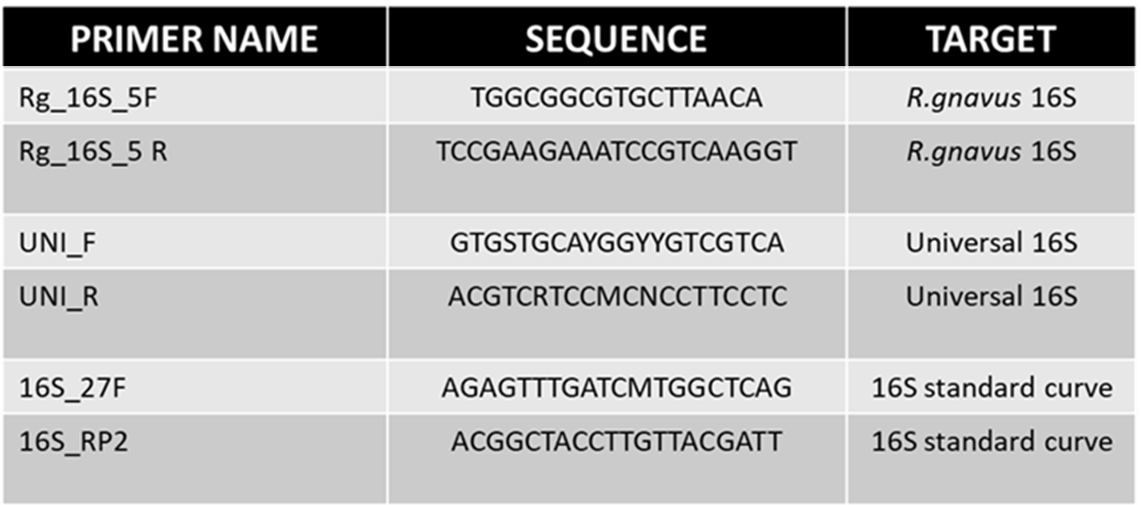


Name and sequence of primers used for qPCR analysis of DNA extracted from fecal samples of mono-colonized mice.

**Table S5. List of mouse primers used for gene expression analysis in isolated hippocampi of *Rg*-mice and GF**


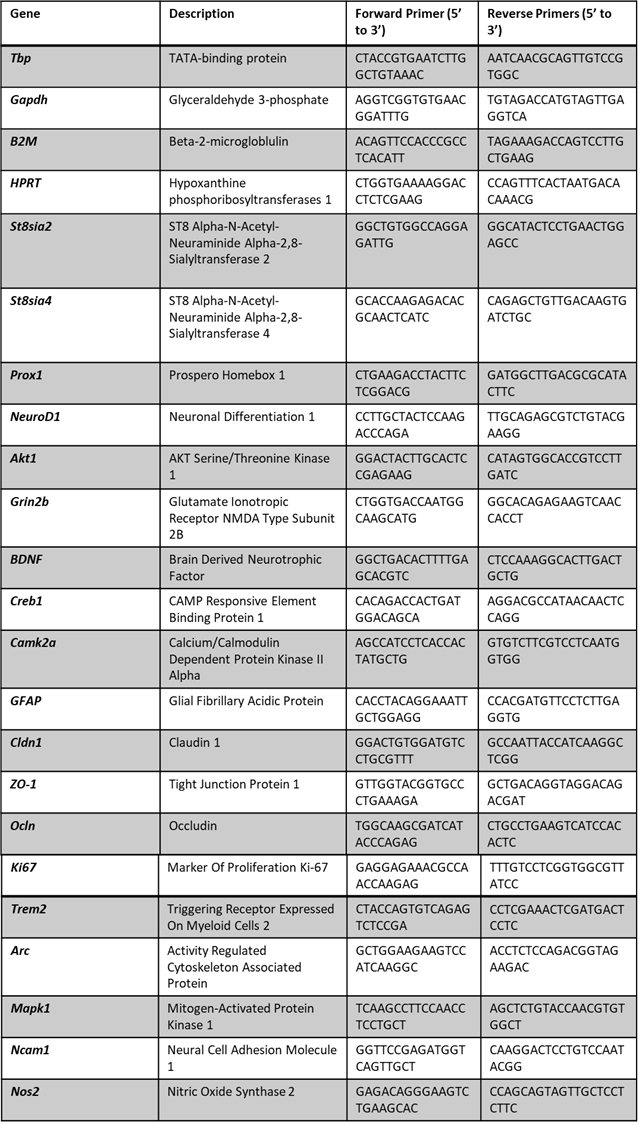


**Table S6. Antibodies used for immunohistochemistry of the murine brain**


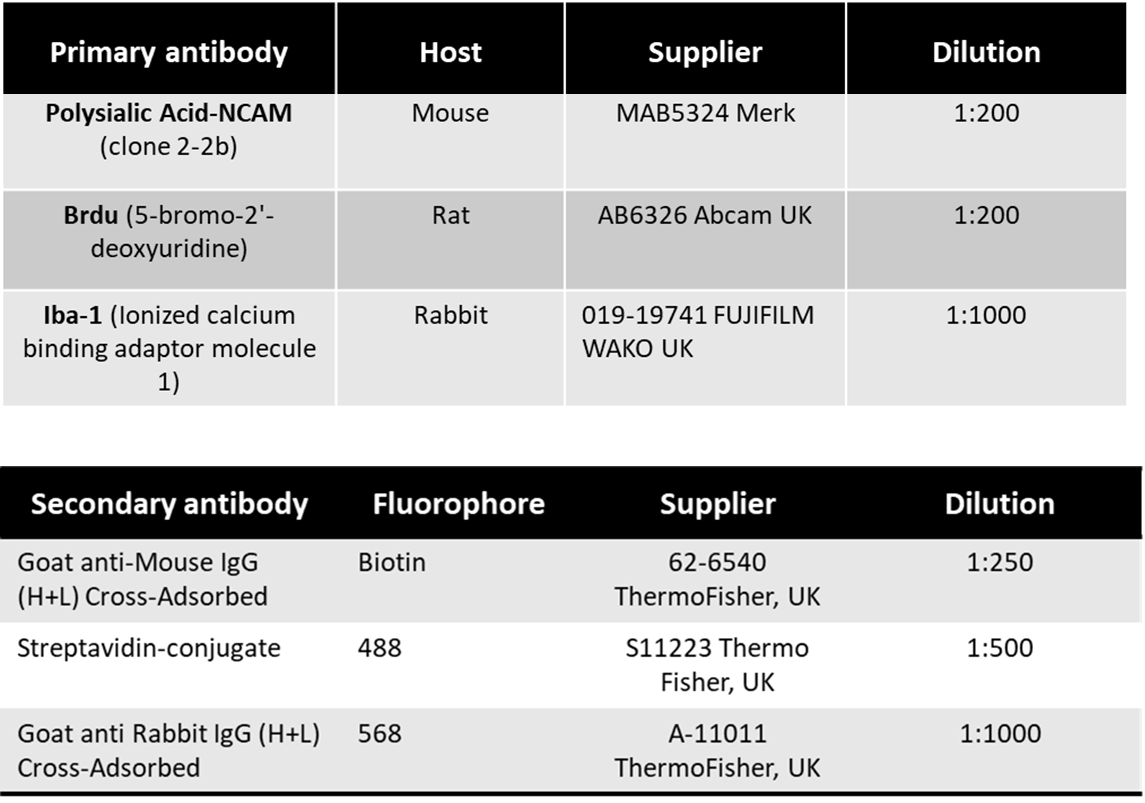


**Figure S1. Level of colonization of *R. gnavus* ATCC 29149 in gnotobiotic mice**


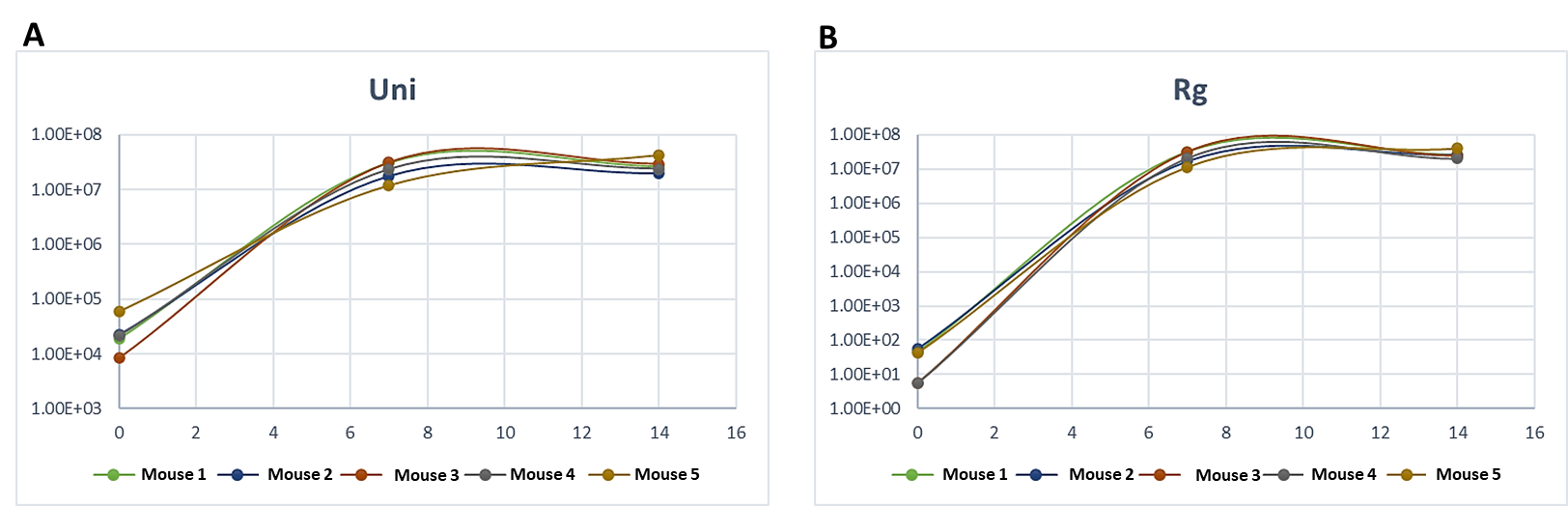


Universal (Uni) 16S primers (**A**) and *R. gnavus* (Rg) 16S specific primers (**B**) were used to monitor colonization in germ-free mice. *R. gnavus* colonization increased from day 0 to day 4, and remained stable until the end of the gnotobiotic study at D14 in all 5 mice. Units are expressed as number of 16S copies/mg of fecal pellet.

**Figure S2. PSA-NCAM immunopositive cells and neurite arborization in the dentate gyrus (DG) of a mouse hippocampus**


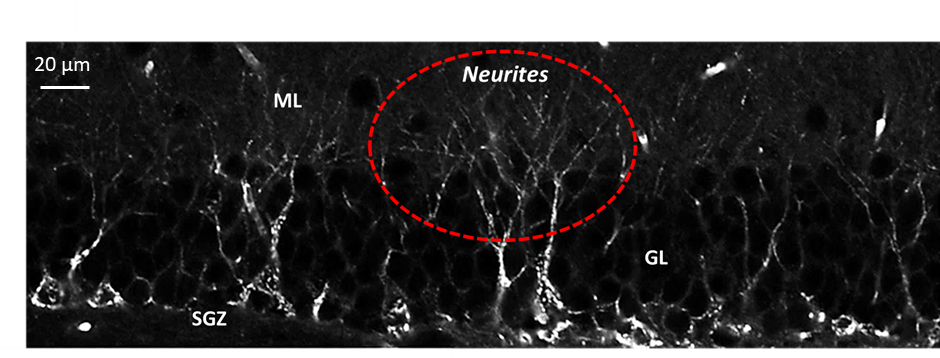


Image of DG region of interest (ROI) for PSA-NCAM analysis. The screenshot shows the area used to manually trace the cell arborizations departing from the PSA-NCAM positive soma cells in the SGZ and spreading upward through the granule cell layer (GCL) toward the molecular layer (ML) of the hippocampus.
